# Street masking: a network-based geographic mask for easily protecting geoprivacy

**DOI:** 10.1186/s12942-020-00219-z

**Published:** 2020-07-06

**Authors:** David Swanlund, Nadine Schuurman, Paul Zandbergen, Mariana Brussoni

**Affiliations:** 1 Department of Geography, Simon Fraser University, 8888 University Drive, Burnaby, BC V5A 1S6 Canada; 2 GIS Program, Vancouver Island University, 900 Fifth Street, Nanaimo, BC V9R 5S5 Canada; 3 Department of Pediatrics, School of Population and Public Health, University of British Columbia, British Columbia Injury Research and Prevention Unit, British Columbia Children’s Hospital Research Institute, F511-4480 Oak Street, Vancouver, BC V6H 3V4 Canada

**Keywords:** Geographic masking, Geoprivacy, Street masking, OpenStreetMap, Geomasking, Osmnx, Donut geomasking

## Abstract

**Background:**

Geographic masks are techniques used to protect individual privacy in published maps but are highly under-utilized in research. This leads to continual violations of individual privacy, as sensitive health records are put at risk in unmasked maps. New approaches to geographic masking are required that foster accessibility and ease of use, such that they become more widely adopted. This article describes a new geographic masking method, called street masking, that reduces the burden on users of finding supplemental population data by instead automatically retrieving OpenStreetMap data and using the road network as a basis for masking. We compare it to donut geomasking, both with and without population density taken into account, to evaluate its efficacy against geographic masks that require slightly less and slightly more supplemental data. Our analysis is performed on synthetic data in three different Canadian cities.

**Results:**

Street masking performs similarly to population-based donut geomasking with regard to privacy protection, achieving comparable k-anonymity values at similar median displacement distances. As expected, distance-based donut geomasking performs worst at privacy protection. Street masking also performs very well regarding information loss, achieving far better cluster preservation and landcover agreement than population-based donut geomasking. Distance-based donut geomasking performs similarly to street masking, though at the cost of reduced privacy protection.

**Conclusion:**

Street masking competes with, if not out-performs population-based donut geomasking and does so without requiring any supplemental data from users. Moreover, unlike most other geographic masks, it significantly minimizes the risk of false attribution and inherently takes many geographic barriers into account. It is easily accessible for Python users and provides the foundation for interfaces to be built for non-coding users, such that privacy can be better protected in sensitive geospatial research.

## Background

Maps are often key figures in academic publications but can lead to privacy violations when they contain sensitive information, such as the home locations of HIV patients or victims of domestic violence. Indeed, Curtis et al. [7] demonstrated over a decade ago that such points in maps can be reverse-engineered into real home locations, thereby exposing private information to the public. It is due this fact that geographers have been developing techniques known as geographic masks since 1999 that allow researchers to publish sensitive points in a representative way without putting any individual’s privacy at risk [2]. For instance, when publishing maps containing points that represent home locations of COVID-19 patients, doing so without using geographic masks may allow others to discover the identity of those patients, constituting a major breach of privacy. Using geographic masks to anonymize those home-address points before publishing the map, however, would safeguard patient privacy while also communicating potential infection hotspots. This article describes a new geographic mask called street masking that uses a highly novel, network-based approach to allow researchers to quickly, easily, and robustly protect privacy in their maps.

Early geographic masks included random perturbation and affine transformations [2, 15]. Random perturbation works by relocating points at random within a buffer of a set radius (such as 250 m). Affine transformations, on the other hand, could include translating, rotating, or scaling point patterns either globally (e.g. transforming the entire point pattern at once) or locally (i.e. dividing the point pattern based on a grid and transforming each cell differently). Unfortunately, both of these masks suffer from critical weaknesses: with random perturbation it is entirely possible that some points will only be moved 1 m, and will therefore not adequately protect privacy, while for affine transformations if an attacker knows the identity of only a few points it becomes possible to re-identify the entire point pattern [2, 12].

The mid-point of geographic masking largely built upon random perturbation and was marked by the inclusion of population density into masking techniques. Kwan et al. [14] introduced the notion of weighted random perturbation, wherein the radius of the buffer used to randomly perturb points was multiplied depending on population density, such that points in less dense areas could be masked further than points in more dense areas. Shortly thereafter, Hampton et al. [12] introduced donut geomasking, which not only used population density to adjust masking distances, but also set a minimum radius to ensure that points were displaced at least a certain distance, greatly improving privacy protection. Another innovation by Hampton et al. worth noting is how they used population density to influence displacement distance. Rather than simply multiplying a pre-defined starting distance based on population density, the authors instead calculated the maximum displacement distance that would be required to achieve a specific level of privacy, known as spatial k-anonymity. Essentially, spatial k-anonymity is a metric that represents how indistinguishable a point is from the background population. For instance, a point is 50-anonymous if 49 other people live closer to the masked point than the individual’s actual, sensitive location.

However, a shortcoming of donut geomasking is that it only estimates k-anonymity using population density within census areas, which are assumed to have homogenously distributed population. When population density is heterogeneously distributed, k-anonymity estimates can vastly over-estimate privacy protection [1]. Recent geographic masks have started to use address data in order to mask points based on a more precise measure of population density, such as the verified neighbor and location swapping masks [18, 22]. Rather than arbitrarily displacing points, as previous masks did, these masks instead relocate points to actual, valid addresses. This has the added benefit of ensuring that points are not relocated into impossible locations, such as parks or lakes. As a result, these masks not only provide stronger privacy protection, but also produce results that are more like the original data than previous generations of geographic masks.

This trade-off between the degree to which privacy is protected and the amount of information that is lost due to masking is a central issue that all geographic masks must grapple with. In fact, the desire to maximize this trade-off has likely been the primary driver for the development of geographic masks. The enabling component for this development, however, has been supplemental data, such as census-based population data in donut geomasking and weighted random perturbation and address points in location swapping and verified neighbor.

Despite the great success to which supplemental data have been used, they nevertheless place an added burden on users who must find, clean, use them. While some may consider this burden irrelevant given the importance of protecting individual privacy and upholding research ethics, the reality is that many researchers forgo geographic masking entirely [11, 13]. In 2014, Kounadi & Leitner found that roughly half of the maps containing sensitive data published in journal articles used no masking whatsoever. A later study found similar results in sexual health journals [11]. One major contributing factor to this is likely a lack of education on the geoprivacy risks of maps. Another, however, is that geographic masking is an onerous task with a lack of accessible tools, poor documentation outside of academic literatures, and dependencies on supplemental data that are not always readily available. While there have been efforts that seek to ameliorate the former two issues [20], there is also a need for strong geographic masks that minimizes the burden posed by supplemental data.

This article takes up this task by introducing a technique called *street masking* that leverages road network data to strongly mask sensitive points. Rather than requiring users to download (and potentially clean, process, and join) population or address data, street masking automatically downloads OpenStreetMap road network data and uses it to intelligently mask points, all while producing results that are competitive with population-based methods. Moreover, street masking also addresses the concern recently raised by Seidl et al. [19] that most geographic masks introduce the potential for false attribution, wherein a map-reader mistakenly attributes a sensitive attribute to an individual living at the masked location. As street masking moves points to nodes in the road network, the possibility of a masked point being falsely attributed to a particular household is greatly minimized. Finally, we have made street masking available as an easily installable Python package.

## Methods

### Data

**Fig. 1:**
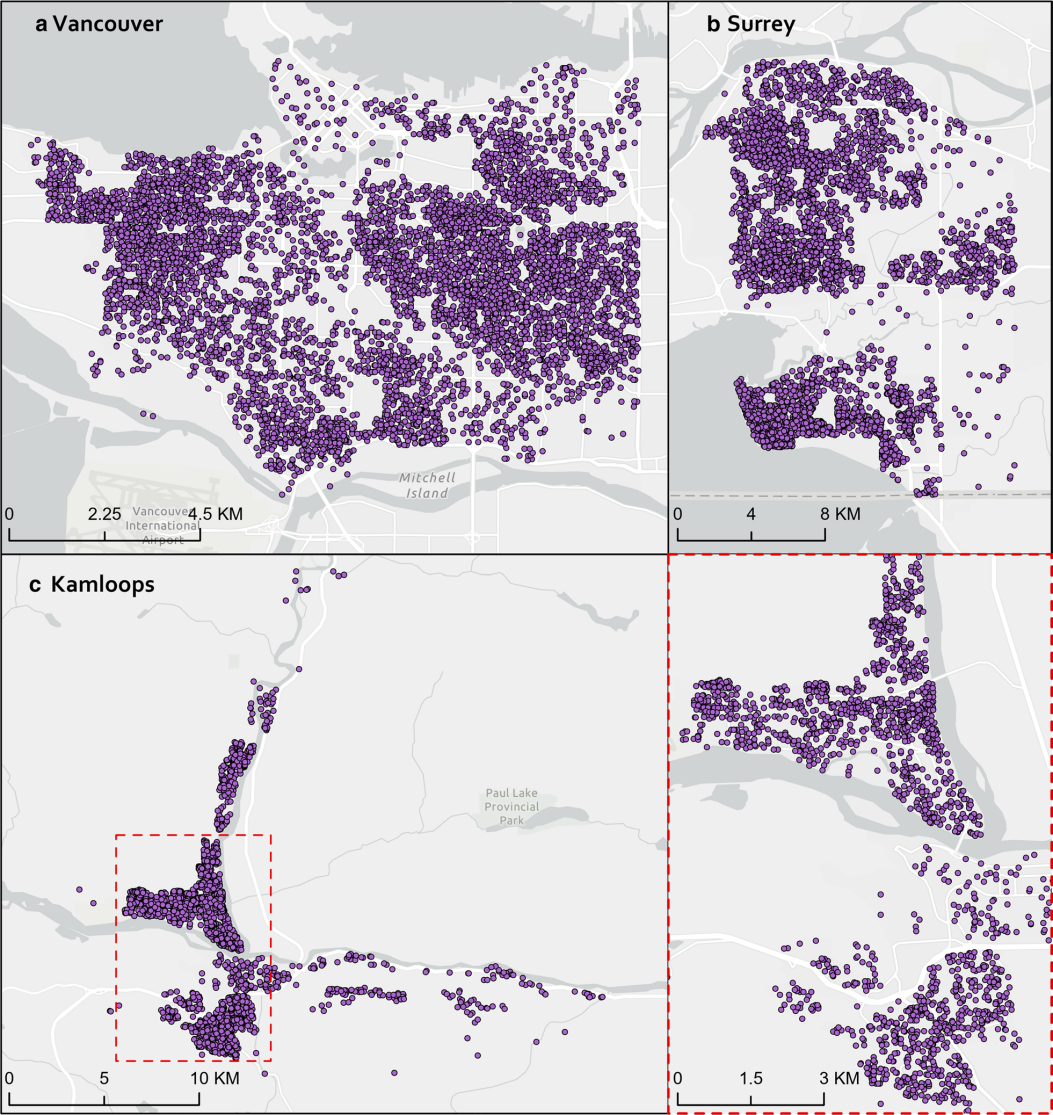
A map depicting the points that were generated in Vancouver (**a**), Surrey (**b**), and Kamloops (**c**, Inset) and used for evaluating each geographic mask

In order to test the street masking method, synthetic data were generated in Vancouver, Surrey, and Kamloops, in British Columbia, Canada. These three cities were selected based on their population characteristics. Vancouver is a major city housing over 630,000 people, with relatively high population density across the city [10]. Surrey is part of the Metro Vancouver region, and has a population of over 550,000. While Surrey’s population is only slightly lower than Vancouver’s, it is much less dense and more heterogeneous, with population centers towards the north, south, and eastern areas of the city, and large agricultural areas occupying its center. Finally, Kamloops is a city in the interior region of British Columbia, with a population of just over 90,000. The population distribution in Kamloops is notable as the city’s physical geography has led to long tendrils of population that grow outward from a central downtown core (Fig. 1). This creates challenges for geographic masking, as points are more likely to be displaced into rivers or mountain-sides than in Surrey or Vancouver where the population is more closely and contiguously distributed.

We chose to create synthetic data for this analysis rather than use actual sensitive health data, which would prevent us from sharing the evaluation data, a common practice in the geographic masking literature [1, 6, 22]. Nevertheless, data can be synthesized to closely simulate a range of potential health events, such as clusters of infectious disease. In this case, we created clusters through a process similar to Zhang et al. [22]. Address points were downloaded from OpenAddresses.io [17], a repository of open address point data. We then categorized the points in each study area into quintiles based on population density from the 2016 Canadian census, before sampling one point from each quintile. This was done in order to create clusters across a range of population densities. Two buffers were created around each sample point with radii of 500 m and 2500 m. A 10% sample was taken within the 500 m buffer, and a 5% sample was taken within the 2500 m buffer. Finally, a 2.5% background sample was taken across the entire study area. This resulted in a total of 7206 address points in Vancouver, 4614 in Surrey, and 2433 in Kamloops, each of which are displayed in Fig. 1. While these data are synthetic, they nevertheless allow us to compare street masking to other established geographic masks, and their clustered nature make them generally representative of many real-world use cases where geographic masking may be used, such as research into the geographic context of geocoded patient data.

### The street masking method

Street masking uses a network-based approach to geographic masking for three primary reasons. First, road network data is more easily retrievable than census or address data, and is also widely available across the globe [3, 16]. If a mask can be developed that can adequately leverage road networks to displace points similarly to population density-based methods, it would be advantageous for simplifying the process of geographic masking. Second, by displacing points to street networks rather than randomly or to other houses, the chances of false attribution are significantly reduced [19]. Lastly, road networks generally conform to geographic barriers such as rivers or cliffs, and so by traversing the network when geographic masking rather than randomly displacing points, these barriers are to a large extent taken into account; a point will not be displaced from the top of cliff to the bottom or from one side of a river to another without a road/bridge connecting them.

**Fig. 2:**
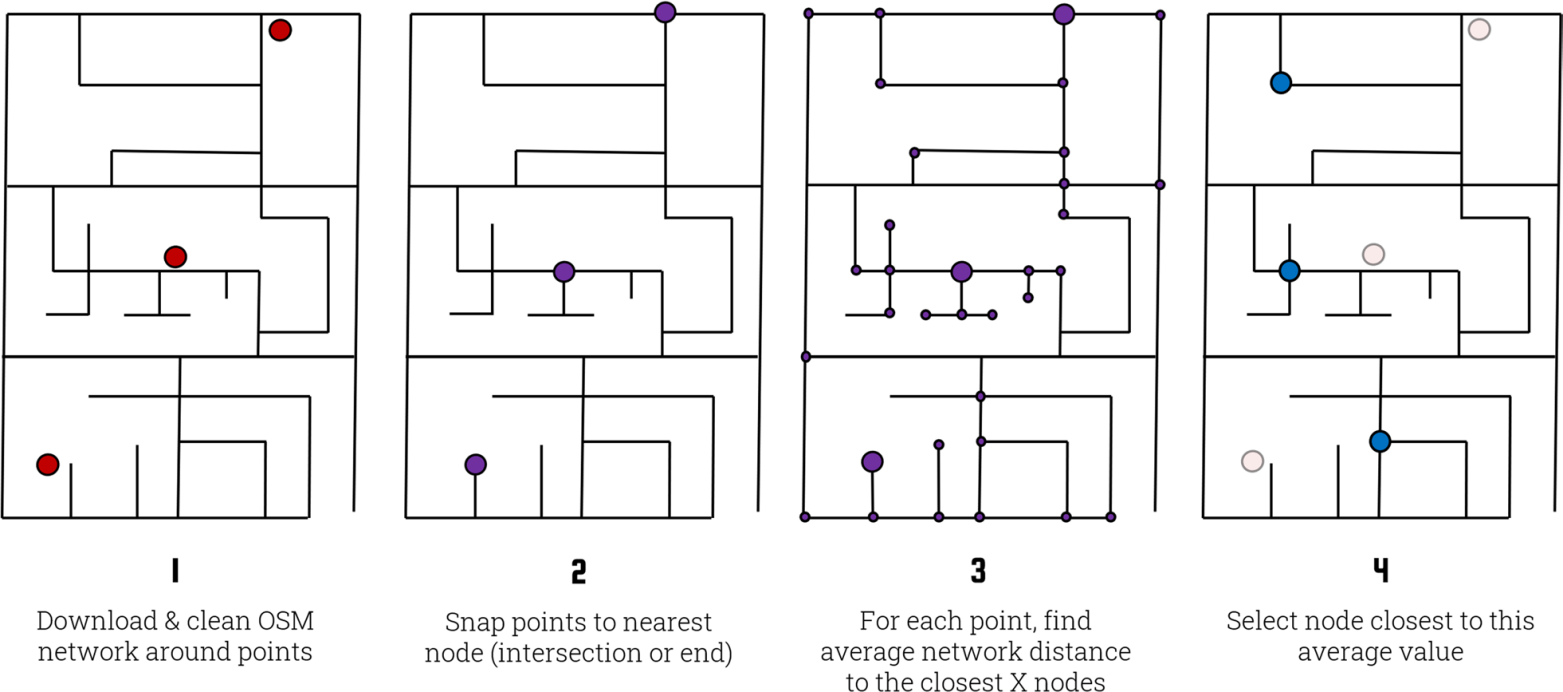
A graphic illustrating the street masking algorithm. Note that the top point is in a low-density area, the middle point is in a high-density area, and the bottom point is in a medium-density area. As a result, the top point is moved the furthest distance after masking, the middle point is moved the least distance, and the bottom point is moved a medium distance

Our street masking method (Fig. 2) begins by first downloading the OpenStreetMap driving network surrounding the input points using OSMnx [5]. OSMnx also cleans the network to remove nodes that have no edges and reduce the number of extraneous nodes “by removing all nodes that are not intersections or dead-ends” [4]. This step is necessary as raw OpenStreetMap data includes many nodes along street segments, particularly as they curve, that would otherwise function as noise in our method. Next, each sensitive point is snapped to the nearest node in the graph (i.e. an intersection or dead-end),^1^ which becomes the starting node. While this provides a base-level of privacy protection, the main purpose of this step is to prepare the data for the main masking process.

The main masking algorithm starts by first building a pool of the closest used-defined-number of nodes (called the search depth value as it controls the depth of the network the algorithm takes into account) based on the network distance (in meters) from the starting node. The average of these distances is then calculated and is used as the target displacement distance. Finally, the algorithm selects a node from the pool whose network-distance from the starting node is closest to the target displacement distance. In other words, the algorithm masks a point by determining the average network-distance to its *x* nearest neighbors and finding the road intersection (node) that is closest to this average value, with the expectation that this average distance would typically be further in rural areas than in urban areas where streets are more dense.^2^

We coded our street masking method into an easy-to-use, parallelized, open source Python package that takes in a search depth value and set of points as a geodataframe (a common spatial format in Python), and then returns the resulting masked geodataframe. It can be easily installed using pip (a package manager for Python) by simply entering pip install maskmypy. This code is also available in the Additional file 1. A Python script for performing street masking, including loading an input shapefile and saving the result, requires only 6 lines of code:
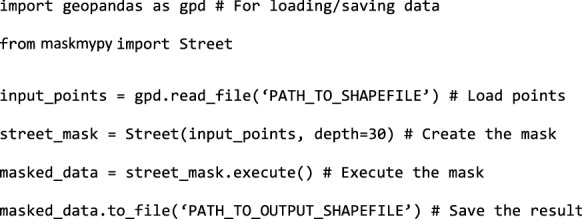


### Evaluation criteria

We chose to evaluate the street masking method against donut geomasking, both with and without population density taken into account. Donut geomasking, as originally described by Hampton et al. [12], randomly displaces points between an outer and inner radius, as determined by a target k-anonymity value and the underlying population density. In other words, instead of selecting a target distance of 300 m, donut geomasking uses a target k-anonymity value, and calculates the displacement distance required to reach that target k-anonymity based on the underlying population density. However, since its publication donut geomasking has also been understood as simply random perturbation with an additional minimum displacement distance (e.g. randomly displace the point between 50 m and 250 m buffers, forming a donut shape) [21]. We refer to the former as population-based donut geomasking, which scales masking distances based on population density, and the latter as distance-based donut geomasking, which uses a single (and relatively subjective) range of potential masking distances. Both provide significantly more privacy protection than ordinary random perturbation, as the minimum displacement distance prevents against points being randomly displaced only 3 m, for instance.

Distance- and population-based donut geomasking were selected as they represent what could be done with slightly less (i.e. no data) and slightly more (i.e. population density) data than is required with street masking, which requires no data from the user and instead automatically retrieves them from OpenStreetMap. To determine the appropriate testing parameters, we performed street masking with a search depth of 10, 20, and 30 nodes within each of our three study areas and determined the median (euclidean) displacement distance for each (Vancouver: 10n = 167 m, 20n = 217 m, and 30n = 271 m; Surrey: 10n = 185 m, 20n = 256 m, 30n = 309 m; Kamloops: 10n = 198 m, 20n = 280 m, 30n = 341 m, where n refers to search depth). We then found parameters for distance- and population-based donut geomasking that resulted in similar (± 5%) median displacement distances. These are listed in Table 1. The minimum displacement distance for each donut geomask was set to 25% of the maximum distance.

**Table 1:** Detailed results of testing each mask based on three different tiers of privacy protection

Mask & parameters			K-anonymity				Displacement distance (m)				Landcover agreement
Area	Mask	Params	K-25	K-50	K-100	K-200	Avg	Med	Min	Max	% of Points
Vancouver, n = 7206	Street	10	94.4	79.7	50.5	16.2	168.8	166.9	38.0	536.3	83.4
	Dist-based donut	225	94.0	82.6	55.0	10.8	168.6	175.2	57.4	225.0	83.9
	Pop-based donut	700	94.9	85.3	52.2	8.0	174.0	160.9	30.5	2814.0	84.9
	Street	20	98.6	93.7	77.5	40.7	220.7	216.6	36.7	720.8	82.7
	Dist-based donut	275	97.2	91.0	73.0	36.6	206.8	215.4	73.0	274.9	82.3
	Pop-based donut	1200	98.0	94.1	80.2	39.3	228.3	210.7	52.9	4259.6	83.0
	Street	30	99.0	96.6	88.2	64.7	270.2	271.1	36.7	949.8	79.7
	Dist-based donut	350	98.6	95.6	86.3	62.3	261.9	270.8	90.5	350.0	81.6
	Pop-based donut	2000	98.9	97.0	91.2	70.7	294.3	271.3	65.2	5018.7	81.4
Surrey, n = 4614	Street	10	90.3	71.0	37.5	7.7	203.5	184.6	37.6	3946.9	90.5
	Dist-based donut	225	82.0	59.2	22.1	0.4	168.8	176.0	59.1	225.0	87.2
	Pop-based donut	500	91.9	73.7	35.2	10.4	280.4	185.3	41.9	7720.1	85.0
	Street	20	97.1	89.3	68.7	30.8	279.4	255.8	44.3	3946.9	88.8
	Dist-based donut	325	92.0	81.4	57.7	21.5	244.6	253.8	83.5	325.0	85.0
	Pop-based donut	1000	96.7	90.7	70.3	30.6	398.8	263.8	55.4	11165.9	81.7
	Street	30	97.7	93.6	81.6	50.1	332.7	309.1	47.2	4138.3	88.4
	Dist-based donut	375	94.1	85.4	67.6	34.7	281.5	293.7	99.5	375.0	83.7
	Pop-based donut	1500	98.1	94.5	83.4	51.5	486.4	321.8	71.2	13731.0	79.4
Kamloops, n = 2433	Street	10	93.3	80.3	51.5	22.0	230.7	198.2	34.1	3901.6	75.7
	Dist-based donut	250	87.1	71.3	39.8	3.7	186.9	195.3	64.0	250.0	73.6
	Pop-based donut	400	93.7	82.4	49.6	16.7	449.8	202.8	45.4	20145.7	66.2
	Street	20	97.7	92.8	77.2	45.7	320.3	280.3	32.6	5123.9	70.9
	Dist-based donut	350	93.5	83.9	64.9	31.6	260.5	270.3	90.6	349.9	68.0
	Pop-based donut	800	97.7	92.6	79.2	45.0	637.5	288.6	63.8	27807.6	63.2
	Street	30	98.7	96.2	87.7	62.9	385.7	340.6	56.3	7020.7	68.6
	Dist-based donut	425	94.8	88.4	75.2	50.3	318.4	332.4	112.2	424.9	64.2
	Pop-based donut	1100	97.7	94.7	86.4	59.7	752.9	339.5	111.8	34597.7	60.8

Parameters are search depth, maximum displacement distance in meters, and maximum k-anonymity for street masking, distance-based donut geomasking, and population-based donut geomasking, respectively

K-anonymity was measured to assess the degree of privacy protection for each mask. Instead of estimating k-anonymity using census-based population data, we used address points and calculated k-anonymity based on these. Specifically, we created a buffer for each masked point with a radius equal to the actual displacement distance from that masked point to its original location. K-anonymity is equal to the number of addresses that fall within this buffer [1]. While these address data do include non-residential addresses, they provide more precise measurements of k-anonymity than census-based estimates, which has been shown to be inaccurate especially when the underlying population distribution is heterogeneous [1]. For each mask, we calculated the percentage of masked points with a k-anonymity equal to or greater than 25, 50, 100, and 200.

Finally, we assessed information loss based on displacement distance, spatial clustering, and landcover agreement. Displacement distance simply refers to the distance between the masked point location and its original location. Spatial clustering was measured to 99% confidence using Ripley’s K function in ArcGIS Pro [9]. Ideally, masked points should exhibit the same level of clustering or dispersion as the original point pattern. We tested clustering across five distance bands using 200 m increments and simulated outer boundary values. Finally, landcover agreement was based on Zhang et al. [22]. We used landcover polygon data from DMTI Spatial [8], which classifies areas according to one of seven landcover types (residential, waterbody, parks, commercial, government and institutional, open area, and resource and industrial), and calculated the percentage of masked points that fell within the same landcover type as their original location. Notably, these landcover types do not include road. As such, landcover agreement functions as another measure of information loss describing how the geographic context of data may change due to geographic masking. Given that many geographic masks displace points completely at random and in doing so may displace points into waterbodies or relocate them from urban areas to rural ones, for instance, we believe landcover agreement to be an important supplementary metric to include beyond just displacement distance and clustering.

## Results

Results (Table 1) indicate that regarding privacy protection, measured using k-anonymity, street masking performs relatively on par with both population- and distance-based donut geomasking in Vancouver. In Surrey, the privacy protection offered by distance-based donut geomasking starts to fall short of the other two masks, while in Kamloops this effect is only made more dramatic. Street masking, however, achieves generally similar k-anonymity values as population-based donut geomasking across all three study areas and at all levels of k-anonymity.

While median displacement distance was used as a control between masks, average displacement distance was highest for population-based donut geomasking, and lowest for distance-based donut geomasking across all masking variations. This was most prominent in Kamloops, where the average displacement distance of population-base donut geomasking was roughly twice that of street masking. Given the similar levels of privacy protection, this suggests that population-based donut geomasking over-displaces many points. This is likely due to points in low-population-density dissemination blocks being displaced extreme distances, despite surrounding blocks being quite dense. This is confirmed in the maximum displacement distances, where population-based donut geomasking displaces points as far as 34.6 km in Kamloops.

Landcover agreement, measured by the percentage of points that are displaced to the same landcover type after masking, was once again similar in Vancouver, but diverged as population densities decreased and population heterogeneity increased in Surrey and Kamloops. In these areas, street masking performed best while population-based donut geomasking performed worst. This is likely due to the fact that donut geomasking randomly displaces points, including into waterbodies or fields, a factor only exacerbated when points were over-displaced by population-based donut geomasking.

**Fig. 3:**
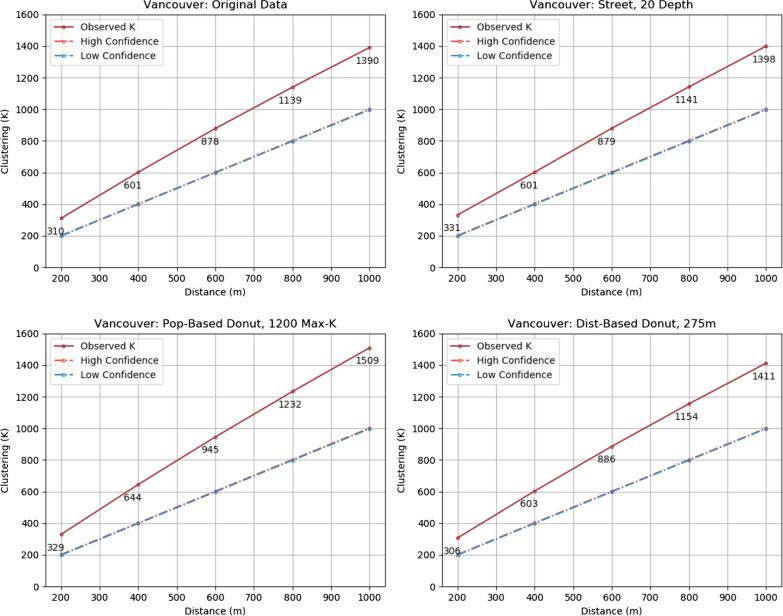
Results of Ripley’s K function analysis for clustering at different spatial scales in Vancouver. Results between the masks are fairly comparable, though population-based donut geomasking tends to increase clustering more than other methods

**Fig. 4:**
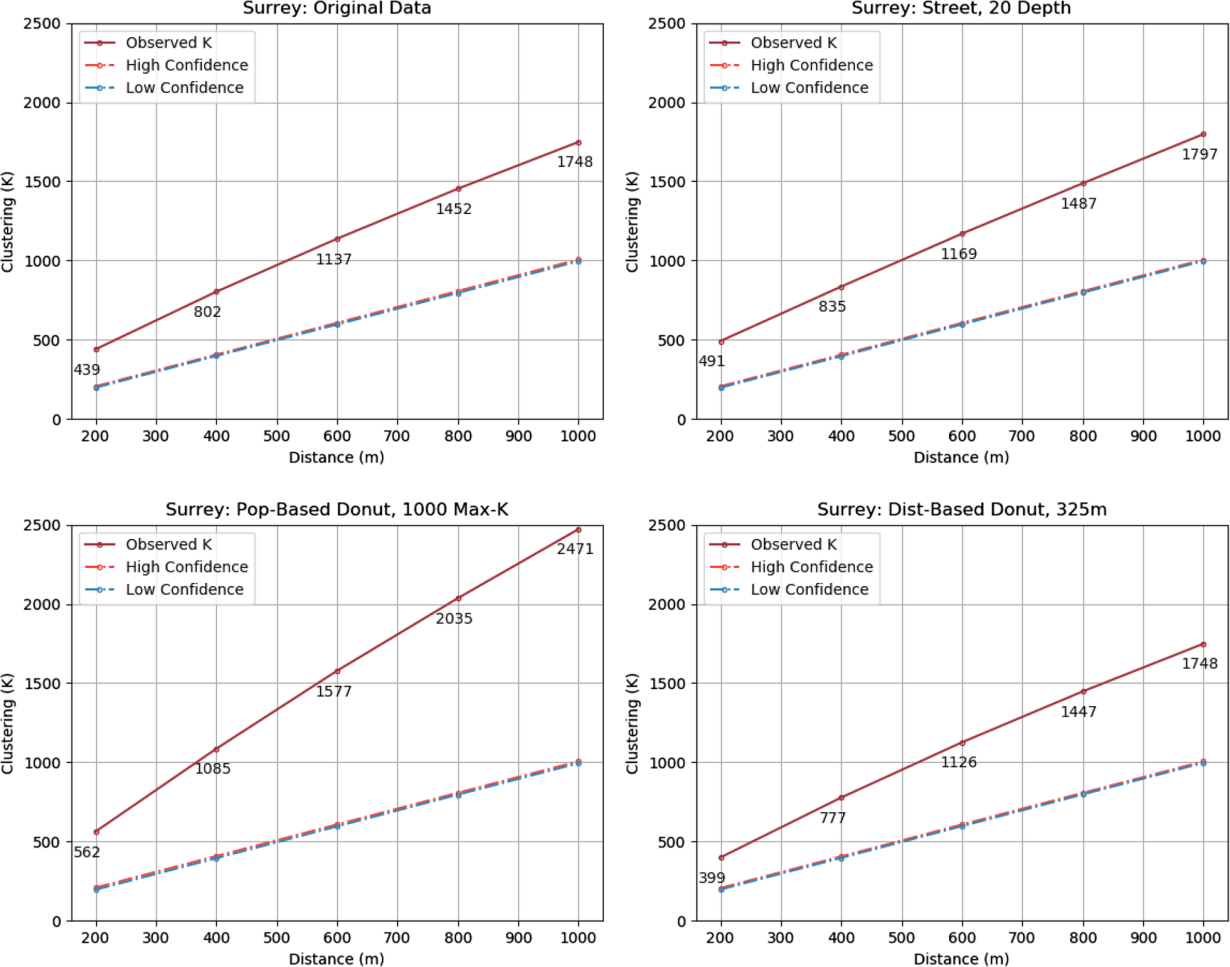
Results of Ripley’s K function analysis for clustering at different spatial scales in Surrey. Results here diverge more than in Vancouver due to increased population heterogeneity. Population-based donut geomasking significantly increases clustering compared to other masks. Street masking and distance-based donut geomasking produce similar results to the original data

**Fig. 5:**
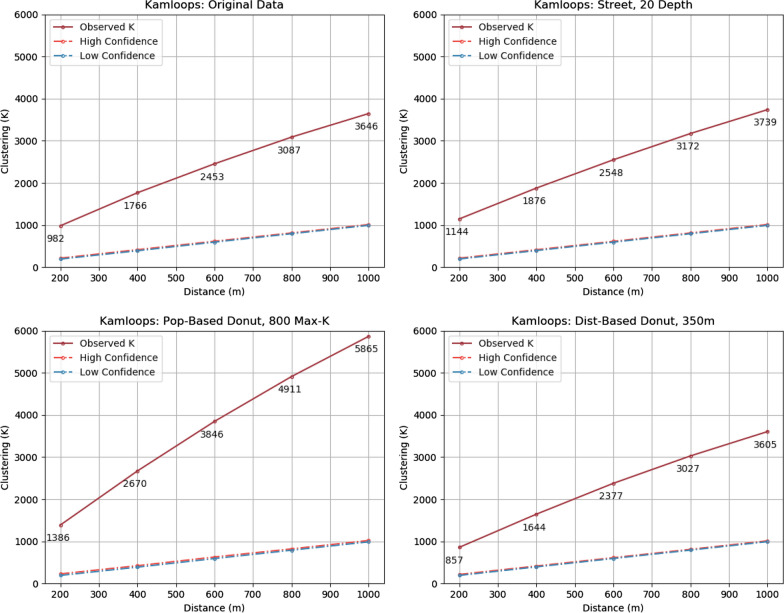
Results of Ripley’s K function analysis for clustering at different spatial scales in Kamloops. With the most heterogeneously distributed population out of the three study areas, results here are most dramatic. Population-based donut geomasking greatly altered clustering. Distance-based donut geomasking is most like the original data, though street masking is not far behind

Spatial clustering is described in Figs. 3, 4, 5. Note that due to the high number of masks that were tested, these figures only describe street masking with a search depth of 20 (i.e. the mid-level) and the equivalent donut geomasks. Clustering graphs for the other masking parameters are available in Additional file 1. As expected, points are highly clustered across all study areas in both the original and masked datasets. Like the previous results, the three masks tended to produce relatively similar results in Vancouver, where population density is highest and most homogeneous. Overall, distance-based donut geomasking tends to generate clusters most like the original data, though once again this comes at the cost of privacy protection as it achieved the lowest k-anonymity values. Population-based donut geomasking, on the other hand, tends to significantly increase the degree of clustering outside of Vancouver, particularly in Kamloops. Finally, street masking preserves clustering nearly as well as distance-based donut geomasking but does so without sacrificing privacy protection.

## Discussion

With street masking’s on-par performance to population-based donut geomasking in Vancouver and strong privacy protection and reduced information loss in Surrey and Kamloops, it is clear that street masking is able to not only compete with but improve on population-based donut geomasking without requiring users to find and load any supplemental data. Indeed, a chief benefit of street masking is ease of use. Whereas other geomasking methods require finding additional population, address, and/or administrative-boundary data in order to intelligently mask points (both in terms of distance and plausible locations), street masking automatically acquires the data it needs for the user. Moreover, as this data were retrieved using OpenStreetMap, they are highly available, including in many low-data environments where supplemental data, address data in particular, can be difficult or impossible to acquire [3, 16].

Another benefit of Street Masking is its ability to largely account for landscape features and geographic barriers. For instance, geographic masks that utilize administrative boundaries to contain points are unable to prevent a point from being displaced across a waterway, cliff, major freeway, or other geographic barrier. With street masking, points are moved according to the road network, helping to preserve topological continuity between original and masked locations.

A recent article by Seidl et al. [19] explores the risk of false attribution when geomasking. This occurs when a map-viewer is unaware that the data have been geographically masked and makes the mistake of assuming that a point over a particular household actually describes that household. While their solution to this was to develop the Voronoi mask, Street Masking potentially provides even greater protection against false attribution as points are displaced to intersections (which may have many homes nearby) rather than property boundaries (which typically straddle only two homes).

Nevertheless, it must be noted that we chose not to compare street masking to address-based masks such as location swapping and verified neighbor masks [18, 22]. This was partly due simply to feasibility of coding and testing all permutations of the masks we already had, but also because we see these masks as having a different use-case. Address-based geographic masking is likely to perform better than street masking but requires data that are often difficult to find or entirely unavailable; street masking, on the other hand, taps into crowdsourced road network data that are highly available and can be automatically acquired, while still providing results that are competitive with population-based donut geomasking. Indeed, geographic masking’s biggest issue is arguably not a lack of adequate methods, but a lack of *adoption* of those methods [11, 13]. We believe street masking is more equipped to ameliorate this adoption problem given its ease of use and efficacy. Regardless, for the most optimal results, address-based geographic masks are likely still the best solution assuming false-attribution is not a concern.

Another drawback is that street masking is not yet easily accessible to those without Python coding skills. While Python is a highly popular language and our method can be executed in a mere 6-line script using entirely open-source tools, an easy-to-use graphical user interface (GUI) is required to make the method more accessible. This is a clear area for future work. Nevertheless, the method and Python package offered in this article are the necessary first steps towards that goal.

Depending on research needs it may also be undesirable for points to be displaced onto roads. In such cases, verified neighbor or location swapping masks should be used. Likewise, street masking likely biases points towards more urban and built landcover types. Depending on the exact landcover data being used (such as highly precise satellite data), this may be problematic, in which case the above masks are again recommended. With our landcover data from DMTI Spatial Inc [8] street masking performed relatively well given its larger (roughly) block-level coverage and lack of roads as a explicit landcover type, whereas with donut geomasking points were more often displaced to entirely different categories.

The final and perhaps most significant drawback of street masking is that the search depth value is not intuitive, and results in differing masking distances as it adapts to the local road network configuration and density. This is of course by design but is also a drawback. Within the three study areas we tested, a search-depth of 30 resulted in relatively strong k-anonymity values, but of course a blanket recommendation cannot be made as this depends on the sensitivity of the data and other factors. Fortunately, the street masking package easily calculates displacement distance, allowing users to explore the distances that result from larger or smaller search depth values in their own study areas. With this information, they can mask their data as easily as distance-based donut geomasking but with far greater efficacy.

## Conclusion

This study developed a method of geographic masking that uses OpenStreetMap road network data to protect privacy, called street masking. By using OpenStreetMap data rather than population data, we were able to produce an open-source Python package that can automatically retrieve the required supplemental data for users transparently and have done so with results that are highly competitive with, if not slightly better than, population-based donut geomasking. In fact, results show that the method improves with increased population heterogeneity, a condition that is typically challenging for geographic masks. Other benefits of the method include that it is inherently able to account for geographic barriers, such as cliffs or rivers that might divide the population, while also minimizing the risk of false-attribution.

Given that the biggest issue in geographic masking right now is arguably a lack of adoption [13], the fact that street masking can achieve these results without requiring any supplemental data from users is a major step forward in geographic masking research. While recent research has sought to make more accessible masking tools [20], these tools are limited by supplemental data requirements. Street masking provides a novel way for coding-users to easily and yet strongly mask their data while also setting a foundation for future, easy-to-use interfaces to be created on top of it that support non-coding users.

## Supplementary information

**Additional file 1:** Additional graphs depicting the results of Ripleys k-function across each study area and mask.

## Data Availability

The synthetic clusters, addresses, and masked data are available as shapefiles in the Open Science Framework repository, https://osf.io/6uqkv. Additional cluster graphs are available in Additional file 1. Landcover data is not shared due to licensing agreements.

## Footnotes

1 Careful consideration was given to the inclusion of dead-ends. In some spinal, suburban street networks, for instance, it is possible that snapping a point to a dead-end would reduce the overall masking distance. However, given that we use the average network distance to the closest X nearest nodes, it is also possible that snapping to a dead-end would increase the masking distance because the distance to other nodes is farther. Given this complexity, as well as the fact that snapping points is only the first step before a much larger displacement step, we decided to include both dead-ends and intersections.

2 Notably, this algorithm evolved significantly throughout development of the street masking method. For instance, we tested using (biased) random walks along the network, setting upper and lower Euclidean distance limits, creating ‘rings’ of nodes based on their node-depth from the starting-node and randomly selecting a node on a target ring, etc. Eventually, however, the method became over-engineered with significant path dependency, and so we restarted with the simpler algorithm described above that, after minor tuning, out-performed all previous attempts.
