# Supplementary material for: Street masking: a network-based geographic mask for easily protecting geoprivacy
Source: Int J Health Geogr. 2020 Jul 6;19:26. doi: 10.1186/s12942-020-00219-z (PMC7336090; doi:10.1186/s12942-020-00219-z)
Supplement: Supplementary file 1 — Additional file 1: Additional graphs depicting the results of Ripleys k-function across each study area and mask. [file 12942_2020_219_MOESM1_ESM.pdf]

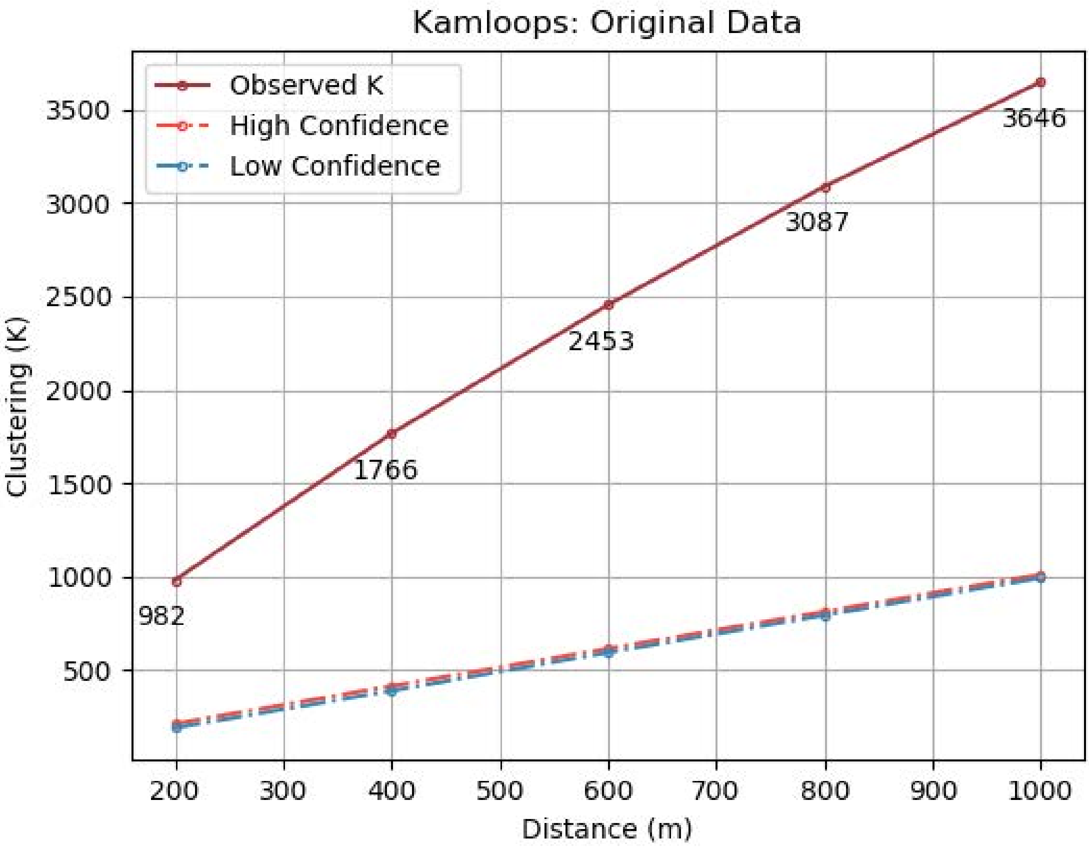

Kamloops: Original Data
Observed K
High Confidence
Low Confidence
3646
3087
2453
1766
982
Clustering (K)
3500
3000
2500
2000
1500
1000
500
200
300
400
500
600
700
800
900
1000
Distance (m)

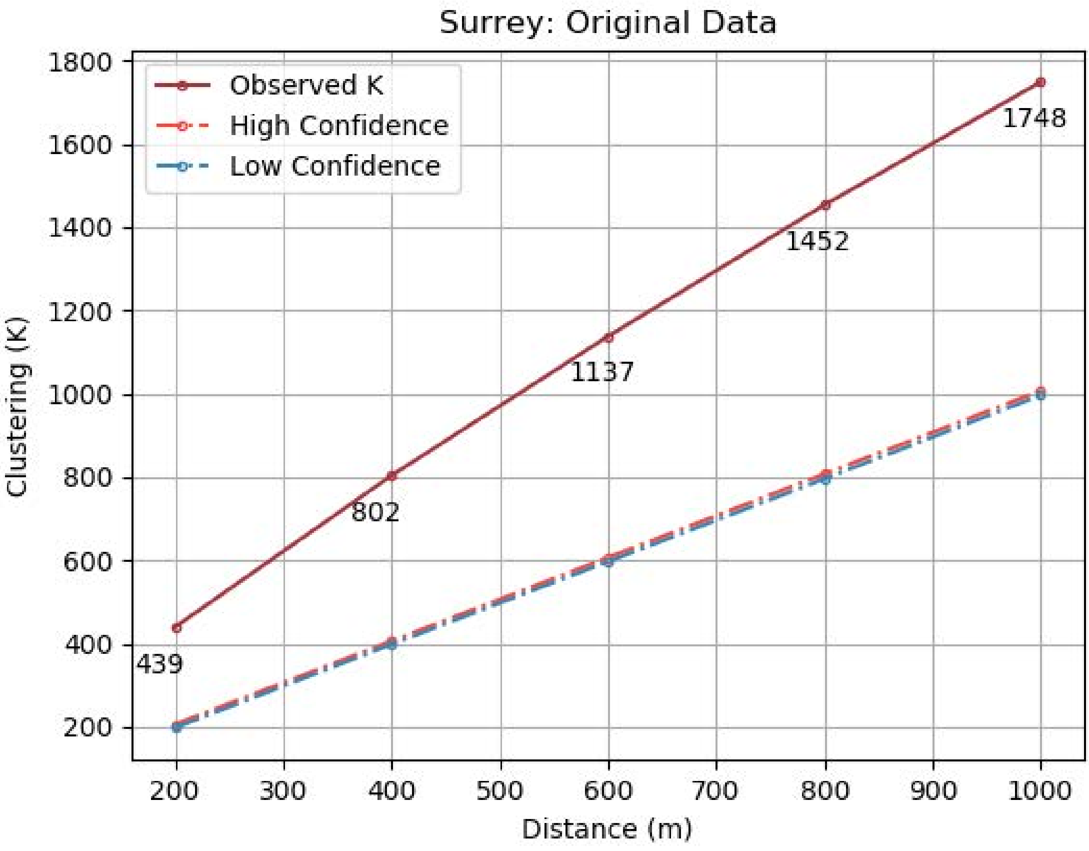

Surrey: Original Data
Observed K
High Confidence
Low Confidence
1748
1452
1137
802
439
Clustering (K)
Distance (m)
200
300
400
500
600
700
800
900
1000
1800
1600
1400
1200
1000
800
600
400
200

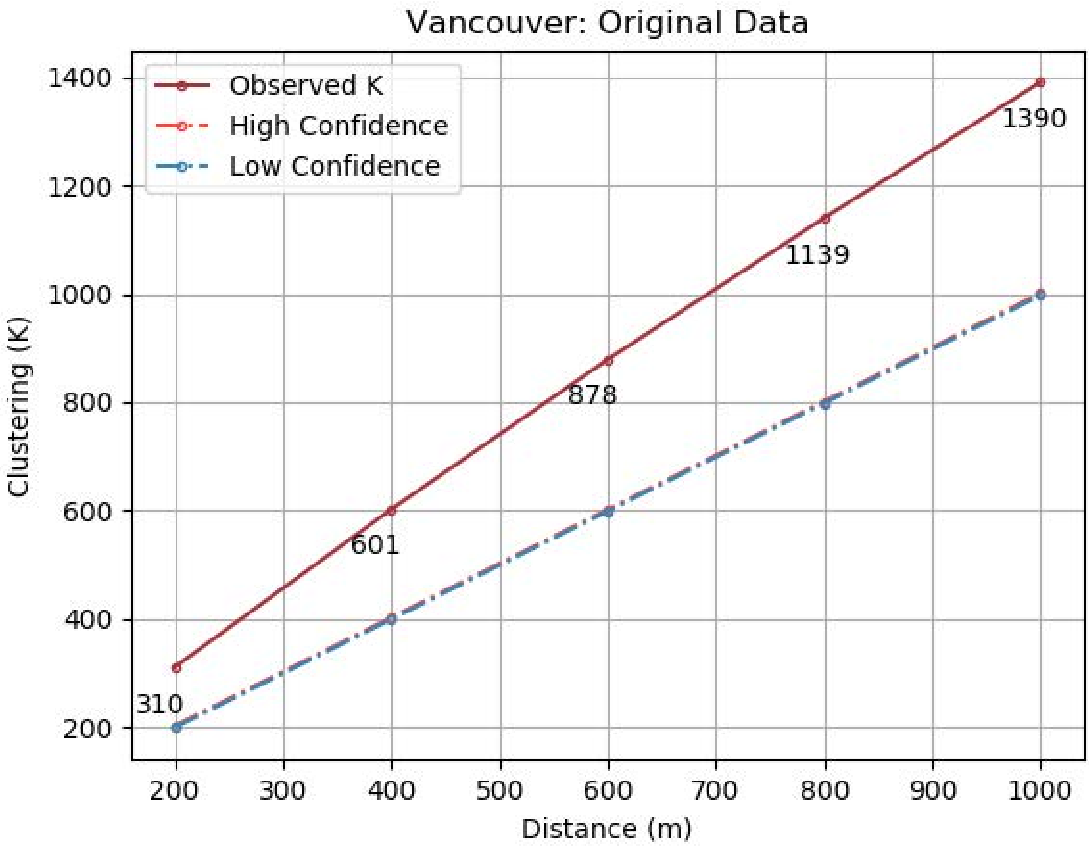
Vancouver: Original Data
Observed K
High Confidence
Low Confidence
1400
1200
1000
800
600
400
200
Clustering (K)
1390
1139
878
601
310
200
300
400
500
600
700
800
900
1000
Distance (m)

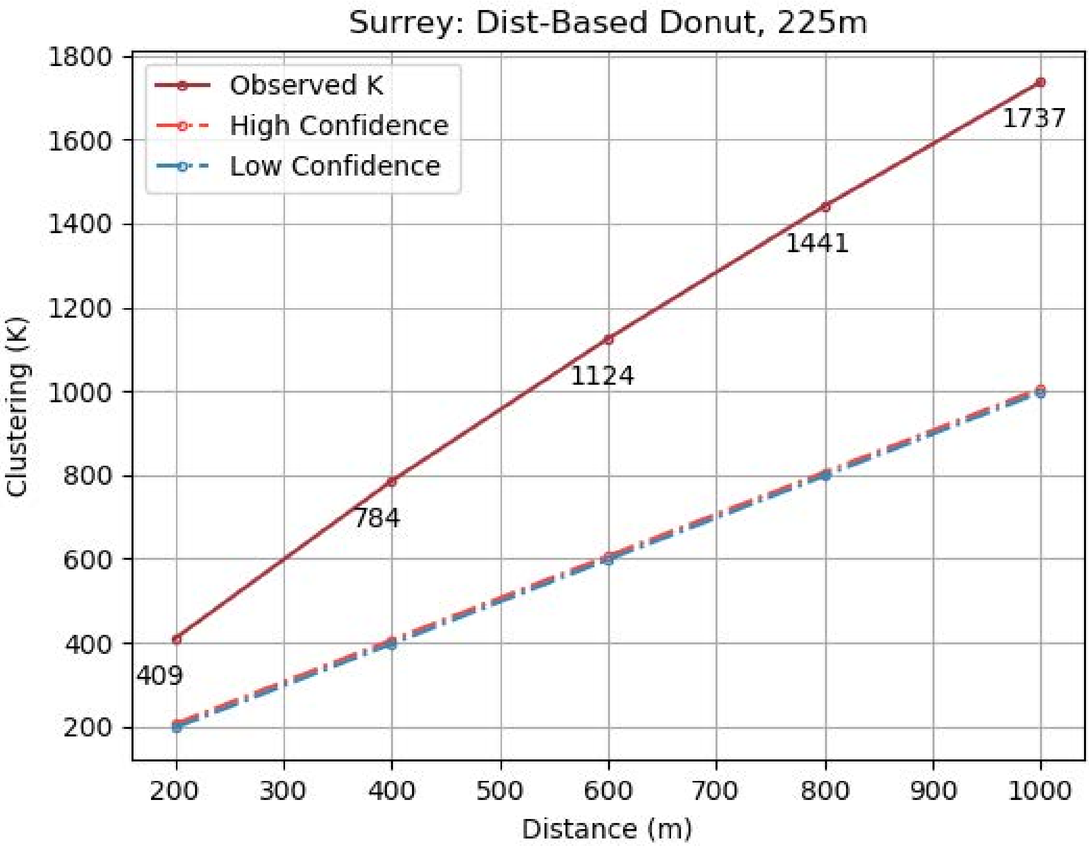
Surrey: Dist-Based Donut, 225m
Observed K
High Confidence
Low Confidence
1737
1441
1124
784
409
Clustering (K)
Distance (m)
1800
1600
1400
1200
1000
800
600
400
200
200
300
400
500
600
700
800
900
1000

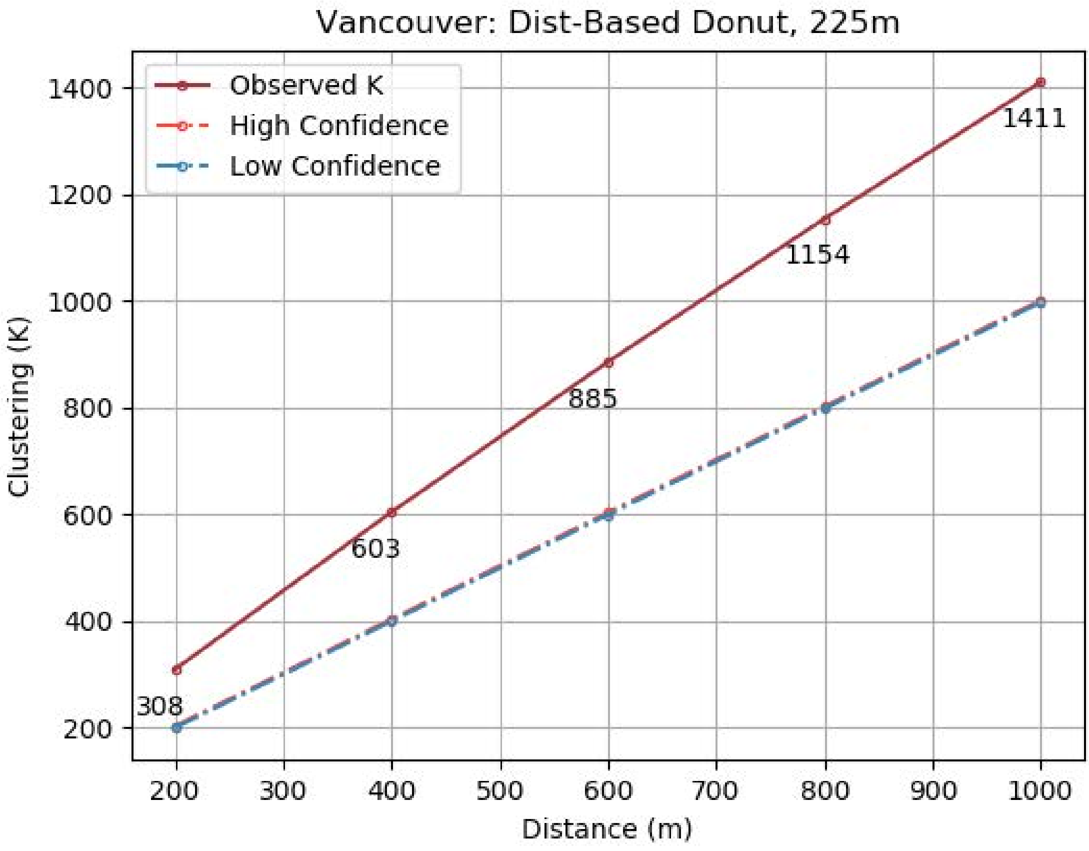

Vancouver: Dist-Based Donut, 225m
Observed K
High Confidence
Low Confidence
1411
1154
885
603
308
Clustering (K)
Distance (m)
200
300
400
500
600
700
800
900
1000
1400
1200
1000
800
600
400
200

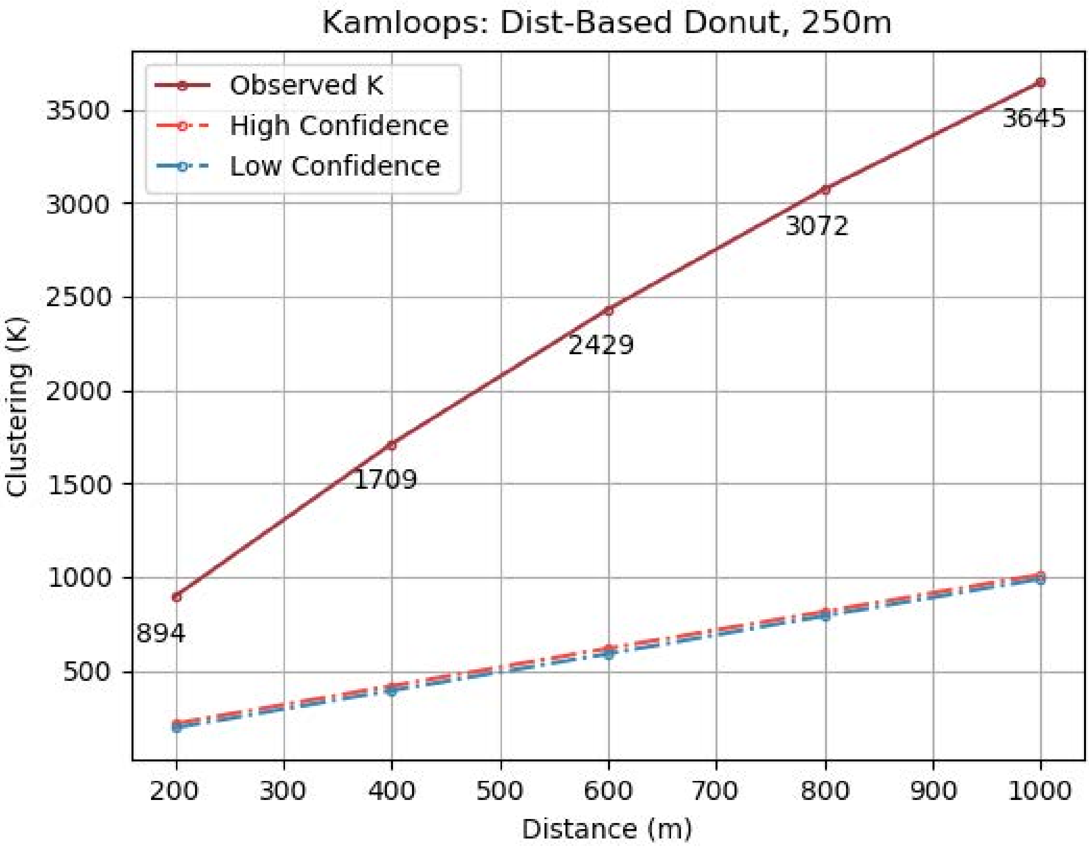
Kamloops: Dist-Based Donut, 250m
Observed K
High Confidence
Low Confidence
3645
3072
2429
1709
894
Clustering (K)
Distance (m)
3500
3000
2500
2000
1500
1000
500
200
300
400
500
600
700
800
900
1000

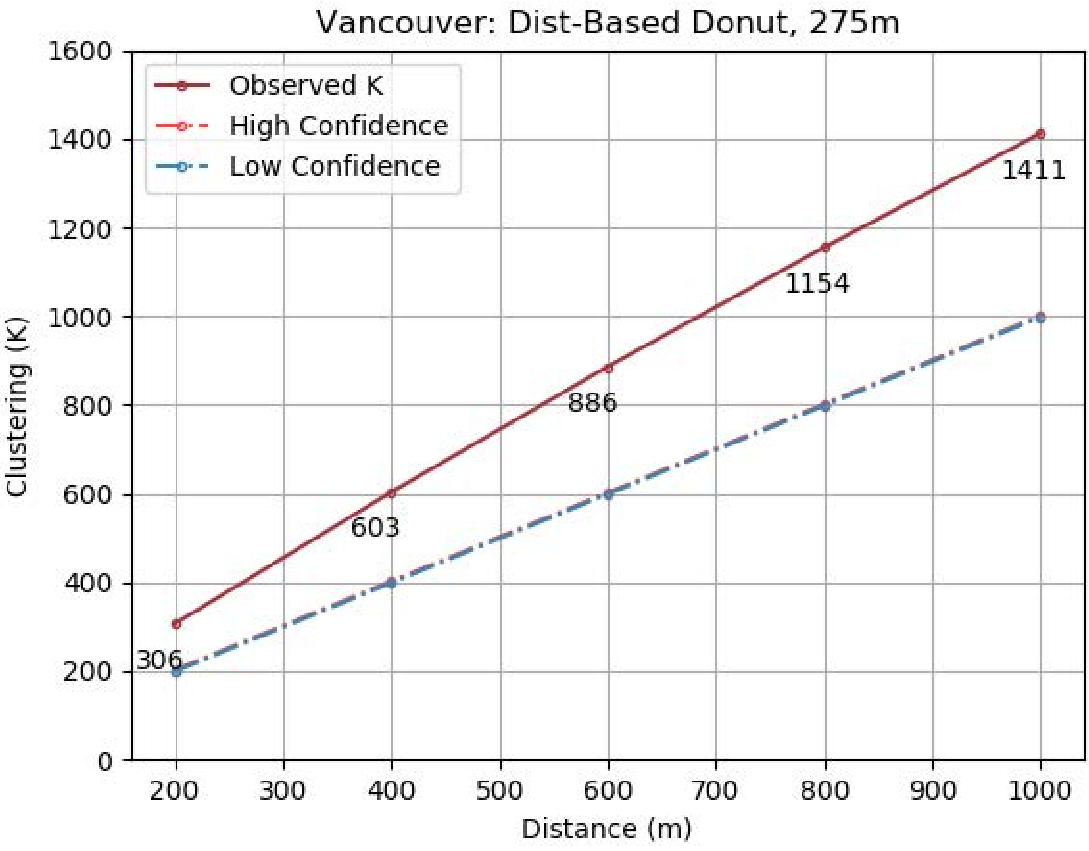
Vancouver: Dist-Based Donut, 275m
Observed K
High Confidence
Low Confidence
Clustering (K)
Distance (m)
0
200
400
600
800
1000
1200
1400
1600
200
300
400
500
600
700
800
900
1000
306
603
886
1154
1411

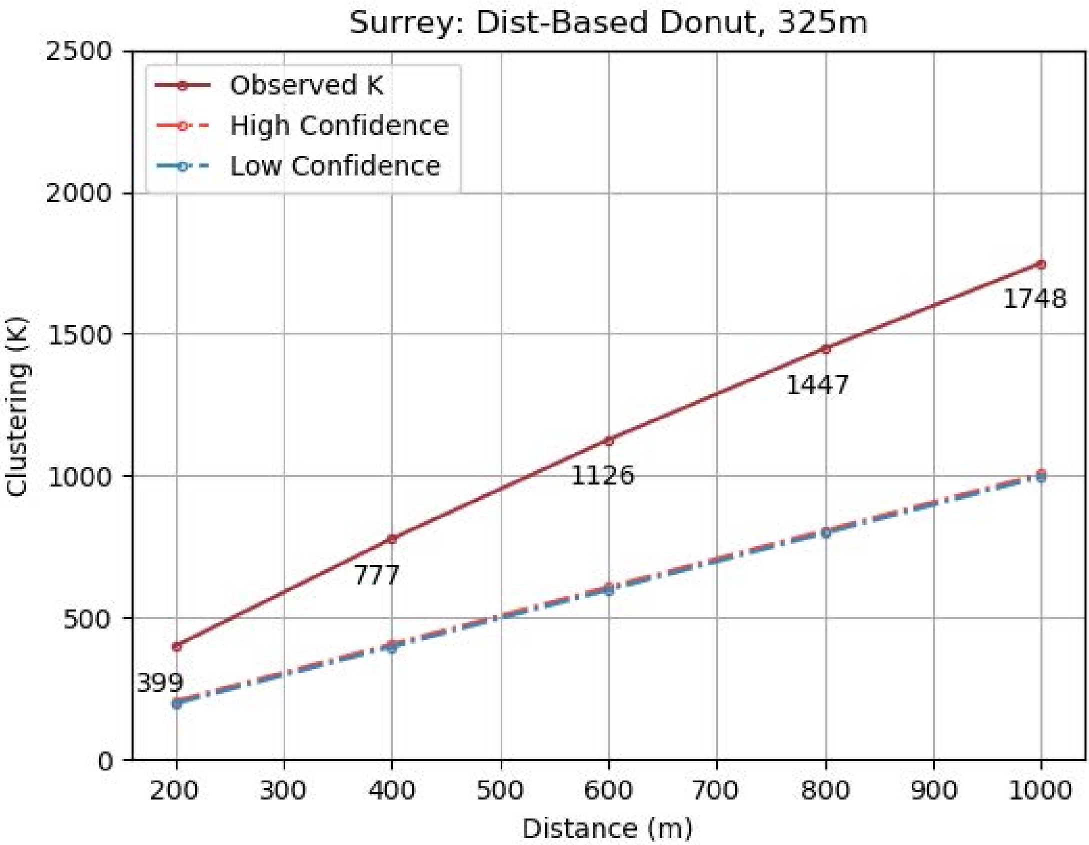

Surrey: Dist-Based Donut, 325m
Observed K
High Confidence
Low Confidence
Clustering (K)
Distance (m)
2500
2000
1500
1000
500
0
200
300
400
500
600
700
800
900
1000
399
777
1126
1447
1748

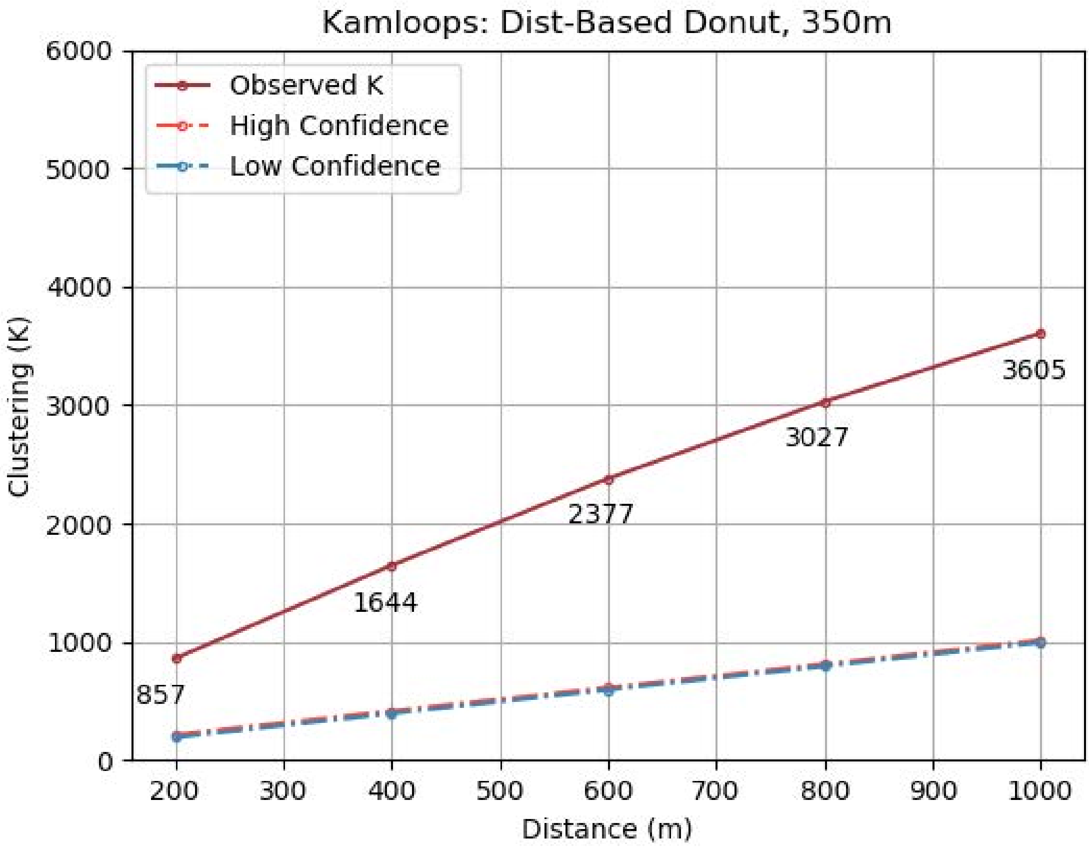

Kamloops: Dist-Based Donut, 350m
Observed K
High Confidence
Low Confidence
Clustering (K)
Distance (m)
6000
5000
4000
3000
2000
1000
0
200
300
400
500
600
700
800
900
1000
857
1644
2377
3027
3605

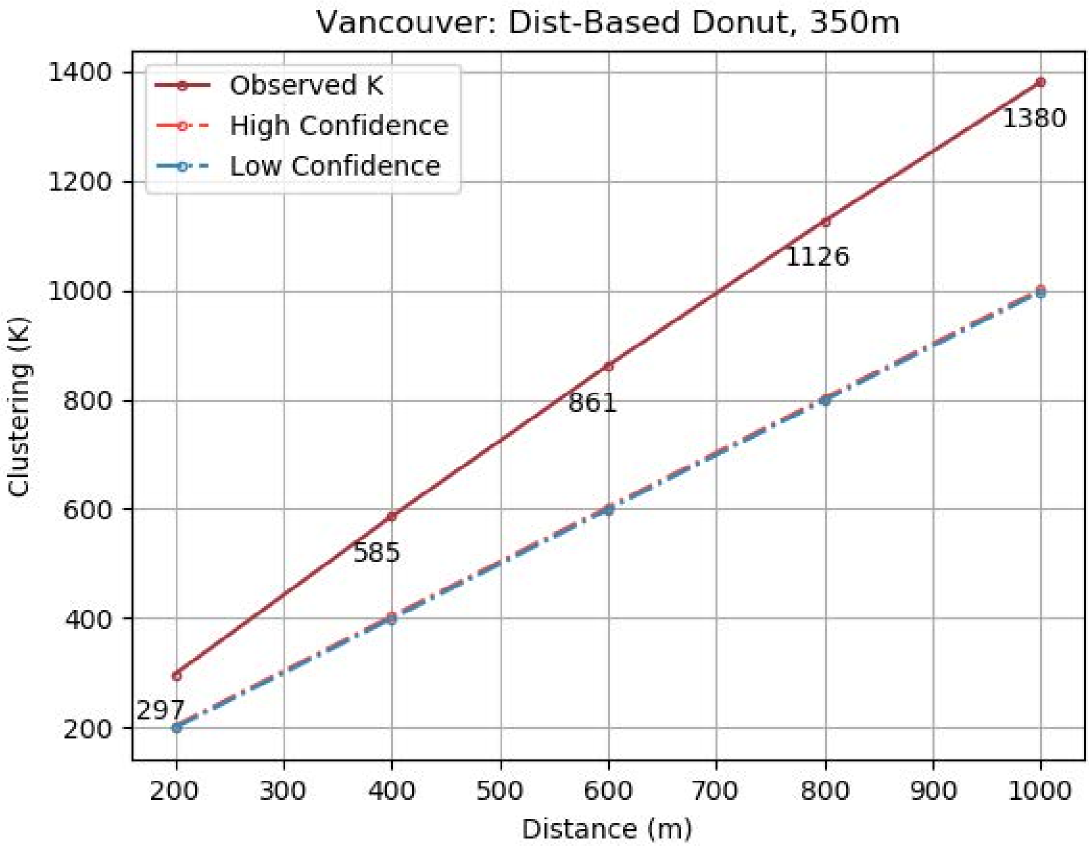

Vancouver: Dist-Based Donut, 350m
Observed K
High Confidence
Low Confidence
1380
1126
861
585
297
Clustering (K)
Distance (m)
1400
1200
1000
800
600
400
200
200
300
400
500
600
700
800
900
1000

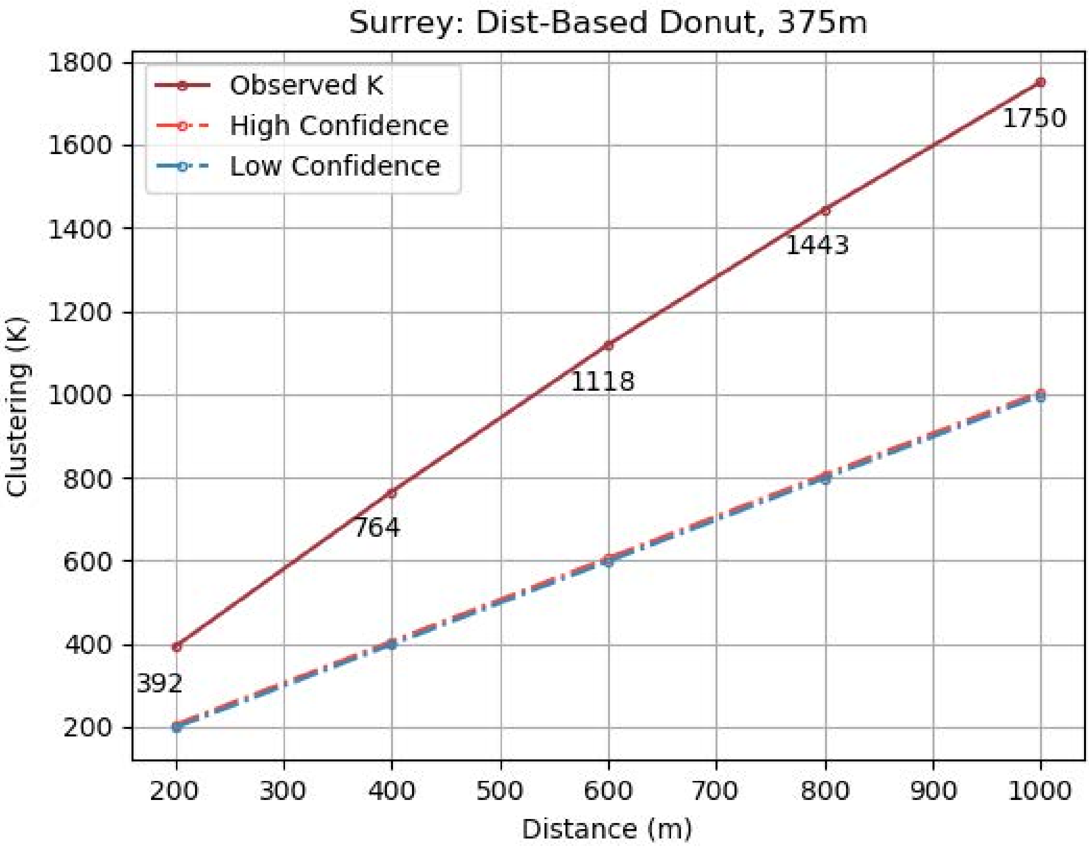

Surrey: Dist-Based Donut, 375m
Observed K
High Confidence
Low Confidence
1750
1443
1118
764
392
Clustering (K)
Distance (m)
200
400
600
800
1000
1200
1400
1600
1800
200
300
400
500
600
700
800
900
1000

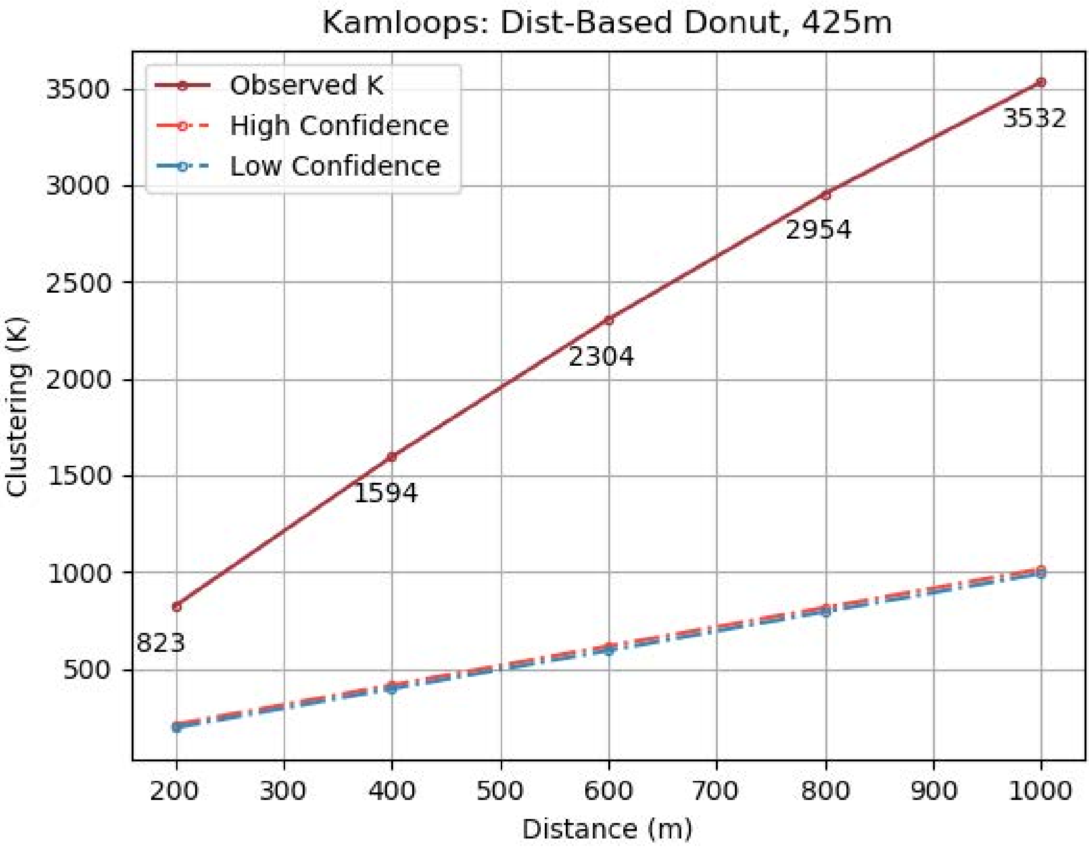
Kamloops: Dist-Based Donut, 425m
Observed K
High Confidence
Low Confidence
3500
3000
2500
2000
1500
1000
500
Clustering (K)
3532
2954
2304
1594
823
200
300
400
500
600
700
800
900
1000
Distance (m)

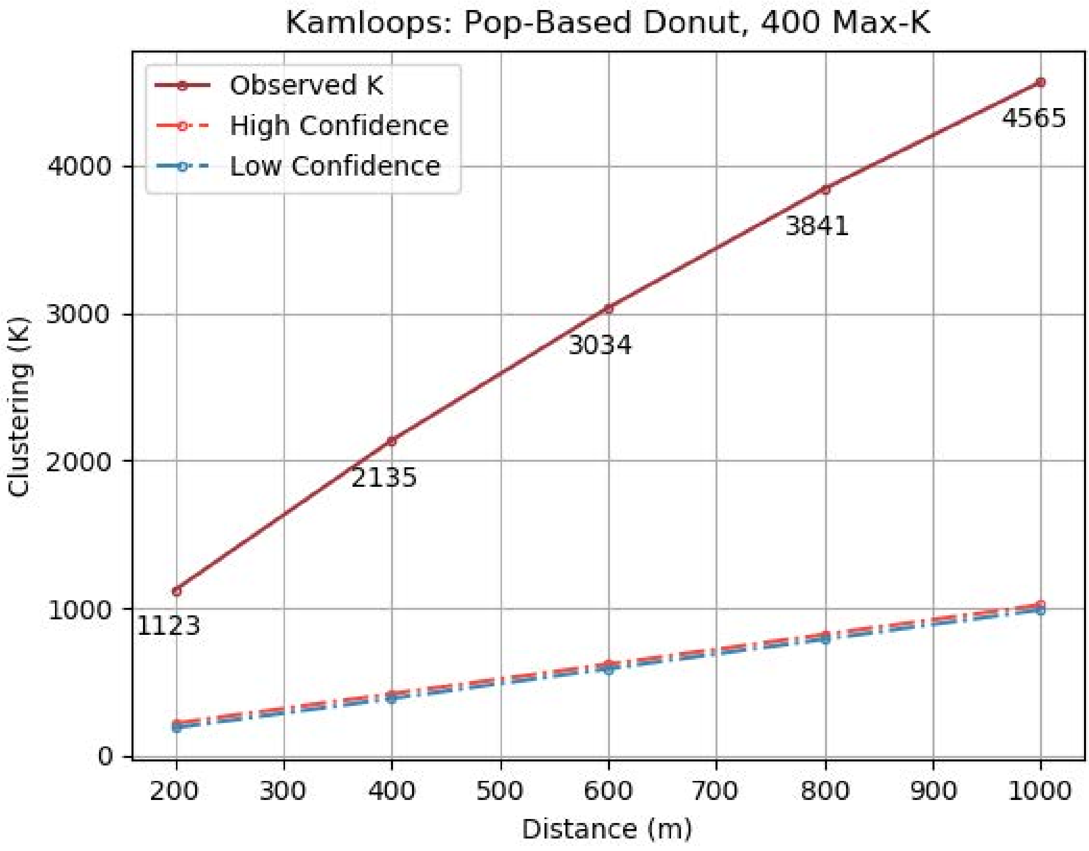
Kamloops: Pop-Based Donut, 400 Max-K
Observed K
High Confidence
Low Confidence
Clustering (K)
Distance (m)
0
1000
2000
3000
4000
200
300
400
500
600
700
800
900
1000
1123
2135
3034
3841
4565

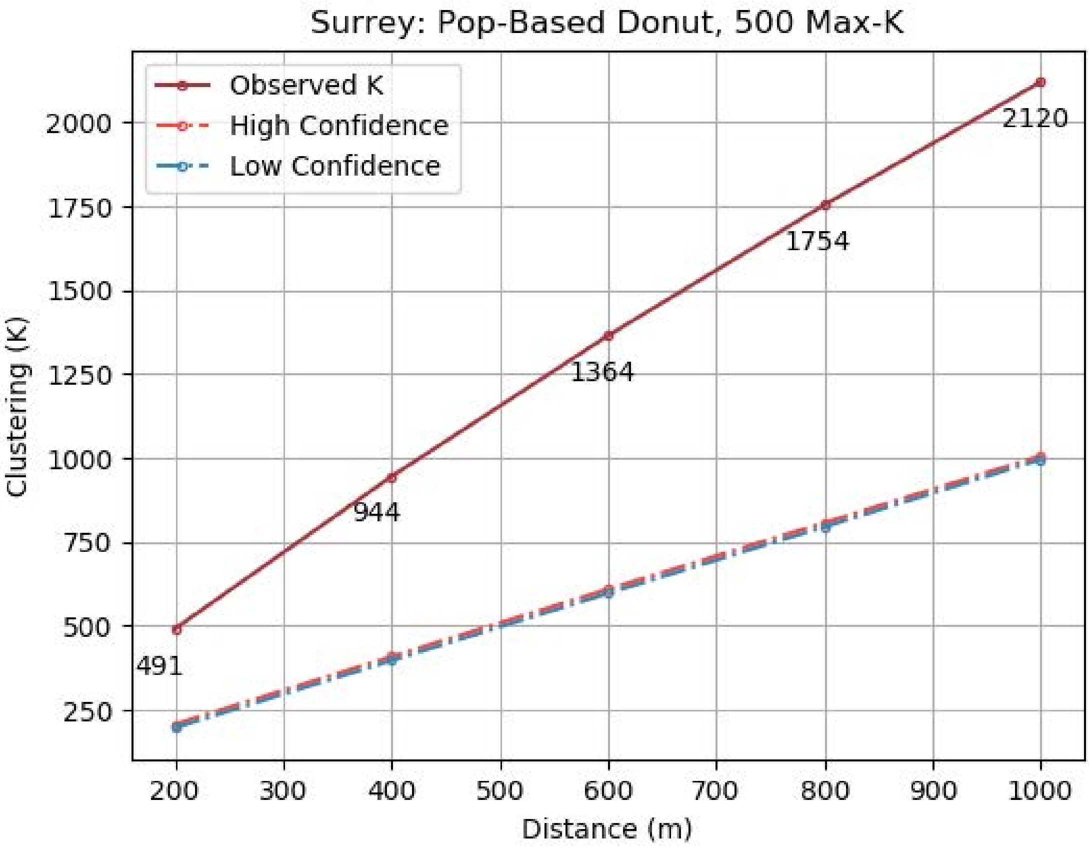

Surrey: Pop-Based Donut, 500 Max-K
Observed K
High Confidence
Low Confidence
Clustering (K)
Distance (m)
491
944
1364
1754
2120
250
500
750
1000
1250
1500
1750
2000
200
300
400
500
600
700
800
900
1000

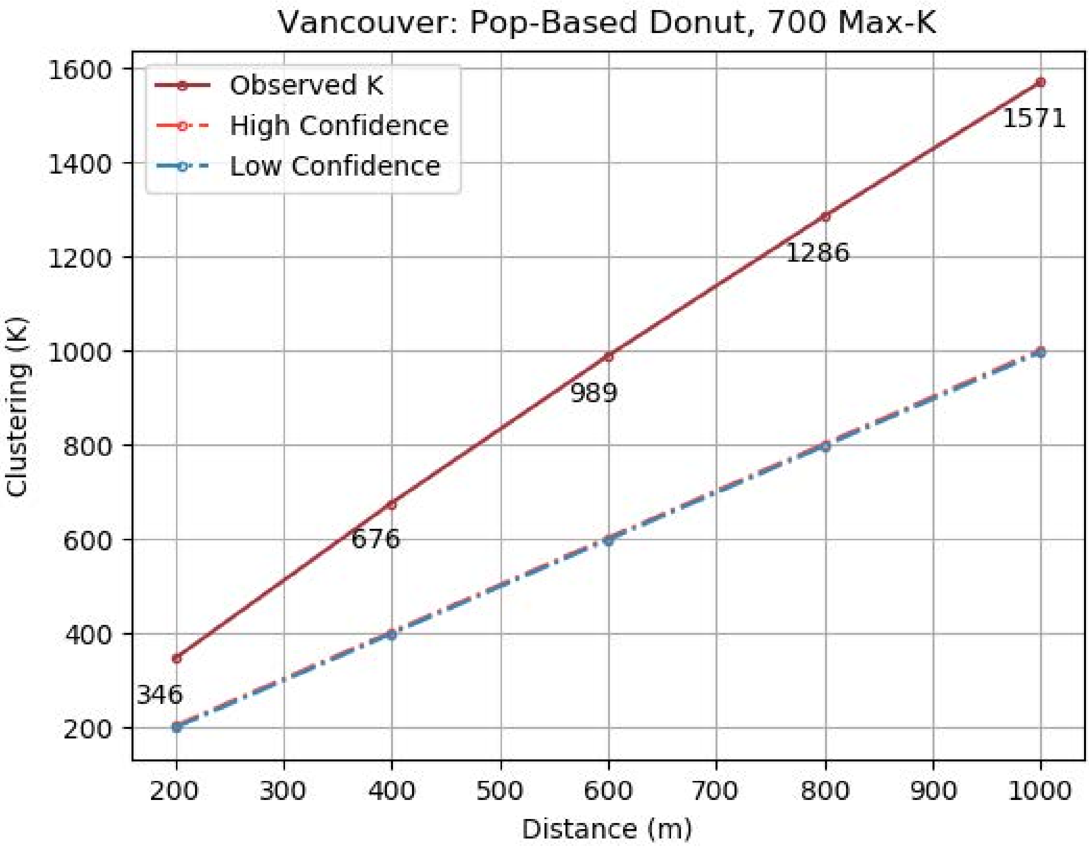

Vancouver: Pop-Based Donut, 700 Max-K
Observed K
High Confidence
Low Confidence
Clustering (K)
Distance (m)
1600
1400
1200
1000
800
600
400
200
300
500
700
900
346
676
989
1286
1571

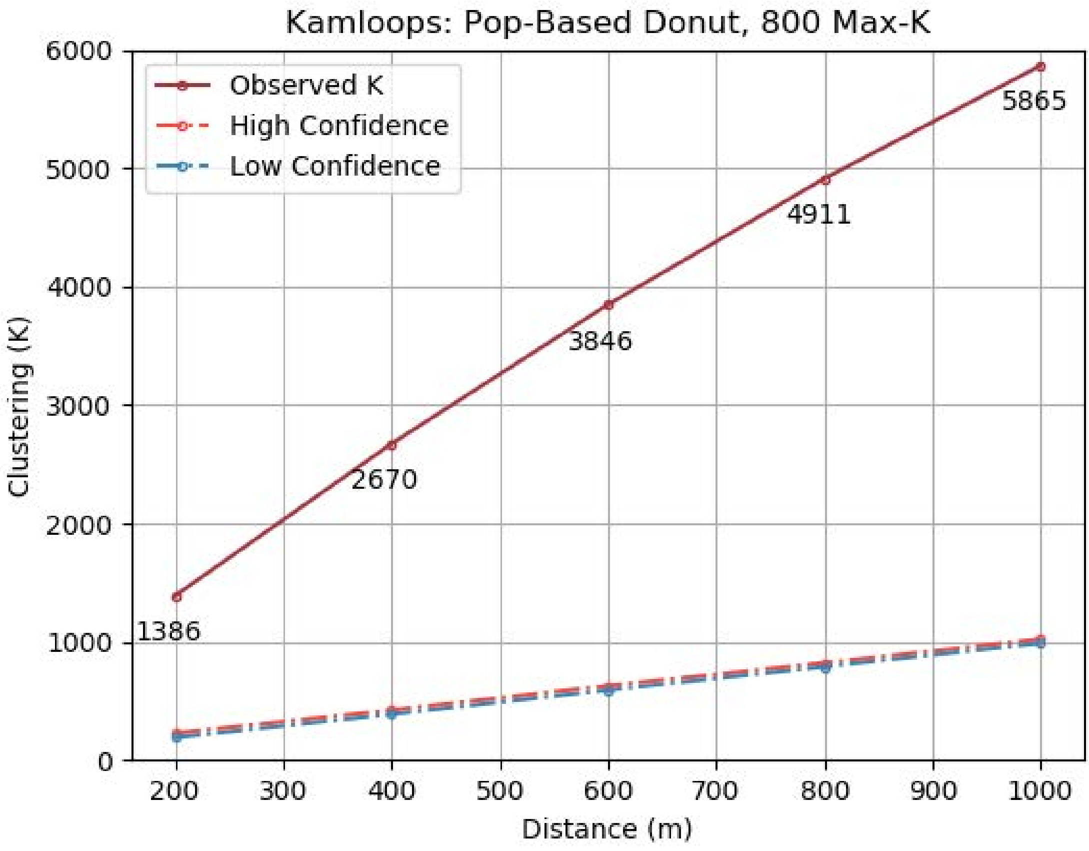
Kamloops: Pop-Based Donut, 800 Max-K
Observed K
High Confidence
Low Confidence
1386
2670
3846
4911
5865
Clustering (K)
Distance (m)
0
1000
2000
3000
4000
5000
6000
200
300
400
500
600
700
800
900
1000

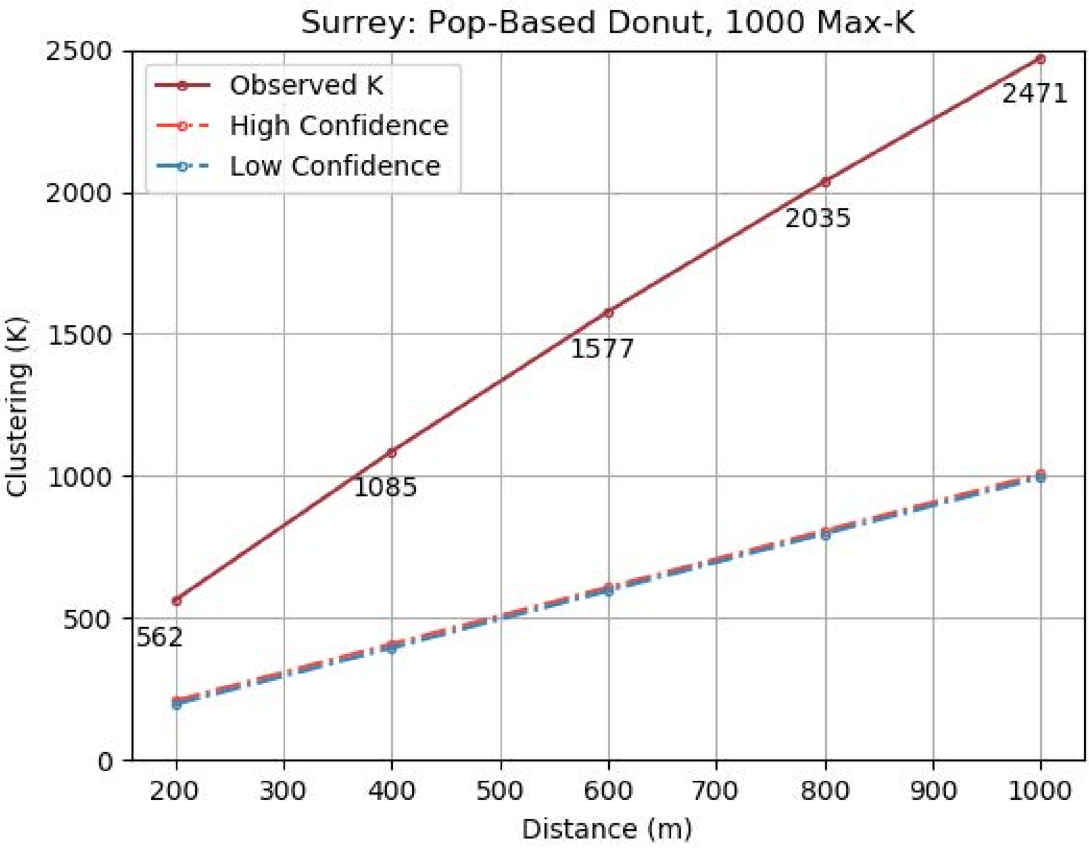

Surrey: Pop-Based Donut, 1000 Max-K
Observed K
High Confidence
Low Confidence
2471
2035
1577
1085
562
Clustering (K)
Distance (m)
0
500
1000
1500
2000
2500
200
300
400
500
600
700
800
900
1000

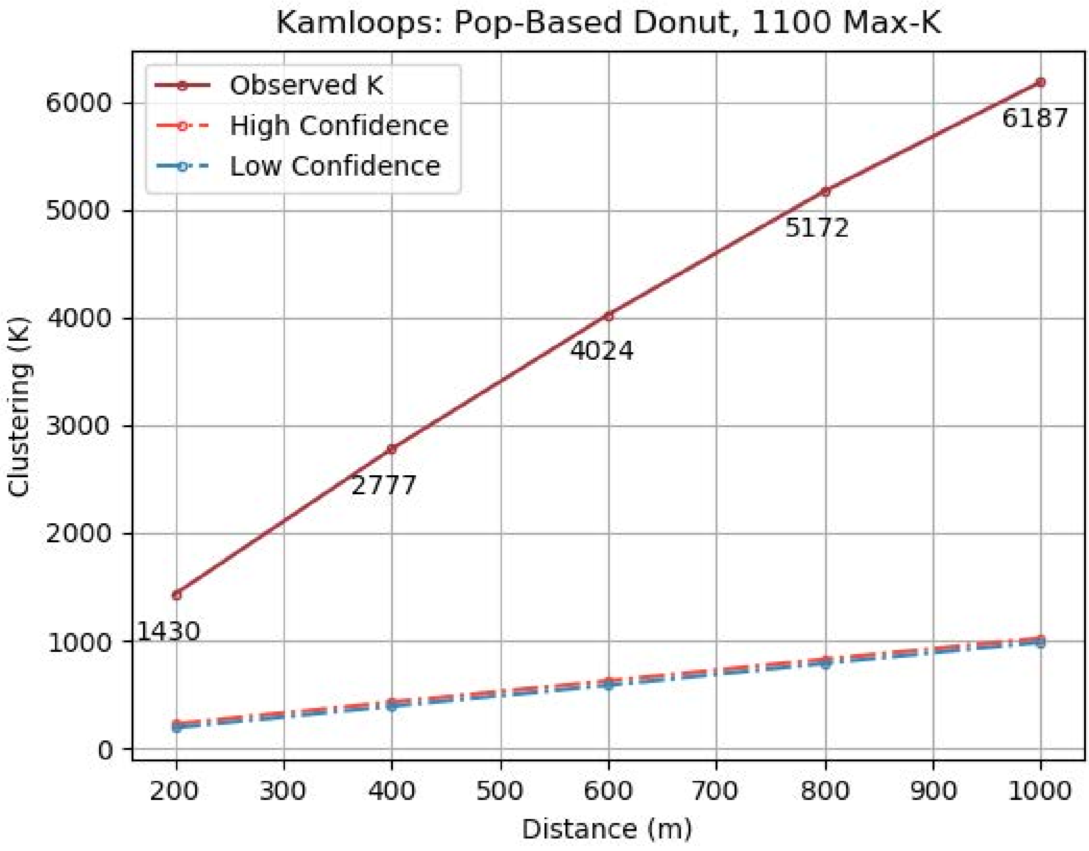

Kamloops: Pop-Based Donut, 1100 Max-K
Observed K
High Confidence
Low Confidence
Clustering (K)
Distance (m)
0
1000
2000
3000
4000
5000
6000
200
300
400
500
600
700
800
900
1000
1430
2777
4024
5172
6187

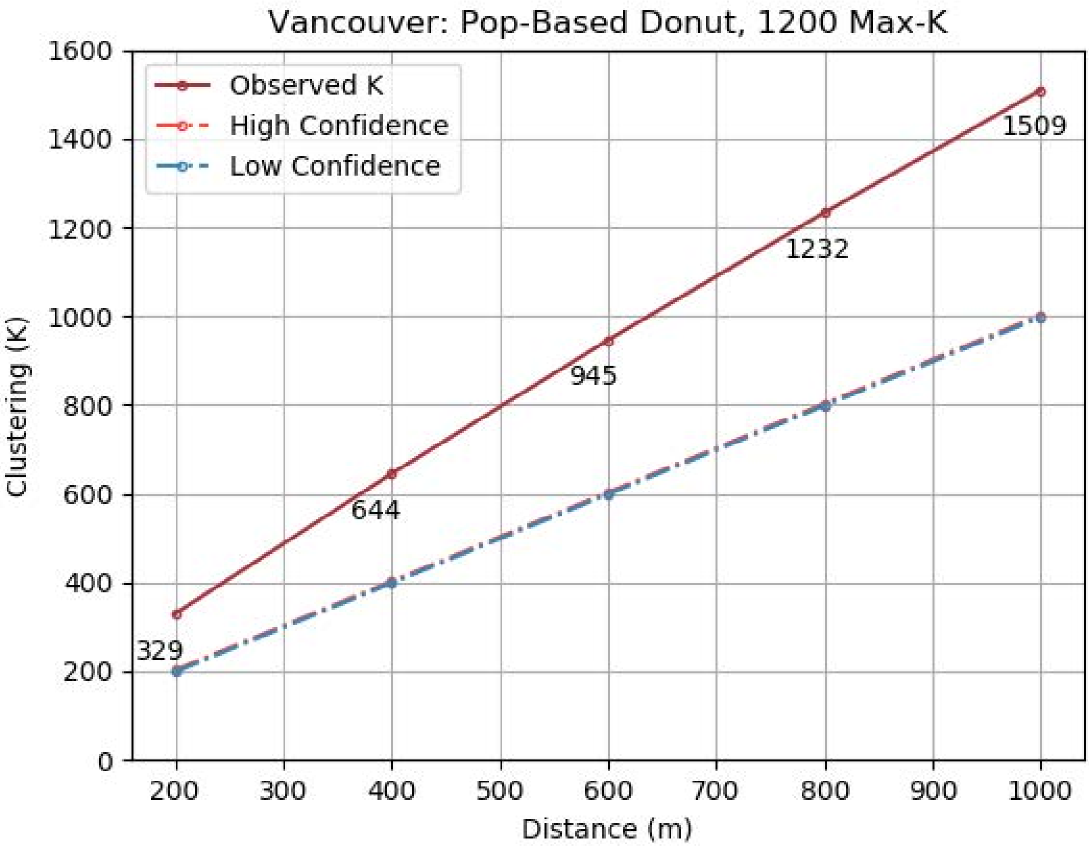

Vancouver: Pop-Based Donut, 1200 Max-K
Observed K
High Confidence
Low Confidence
Clustering (K)
Distance (m)
0
200
400
600
800
1000
1200
1400
1600
200
300
400
500
600
700
800
900
1000
329
644
945
1232
1509

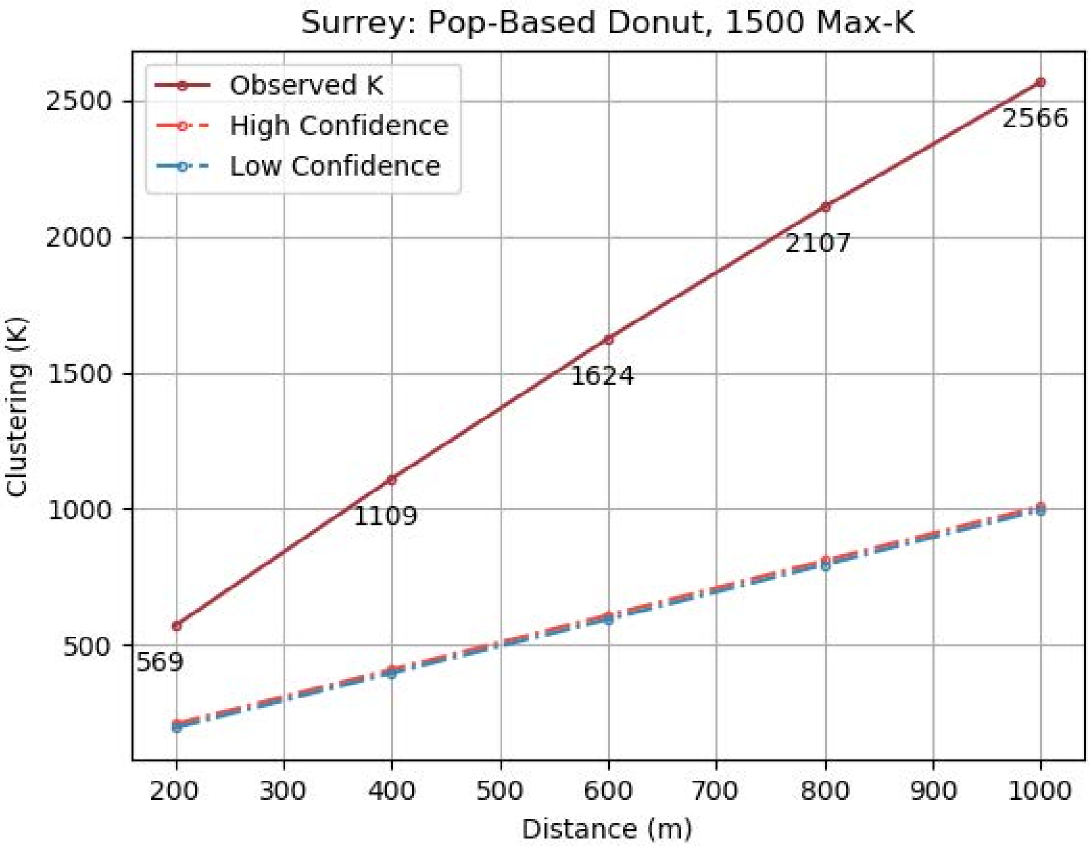

Surrey: Pop-Based Donut, 1500 Max-K
Observed K
High Confidence
Low Confidence
2566
2107
1624
1109
569
Clustering (K)
500
1000
1500
2000
2500
200
300
400
500
600
700
800
900
1000
Distance (m)

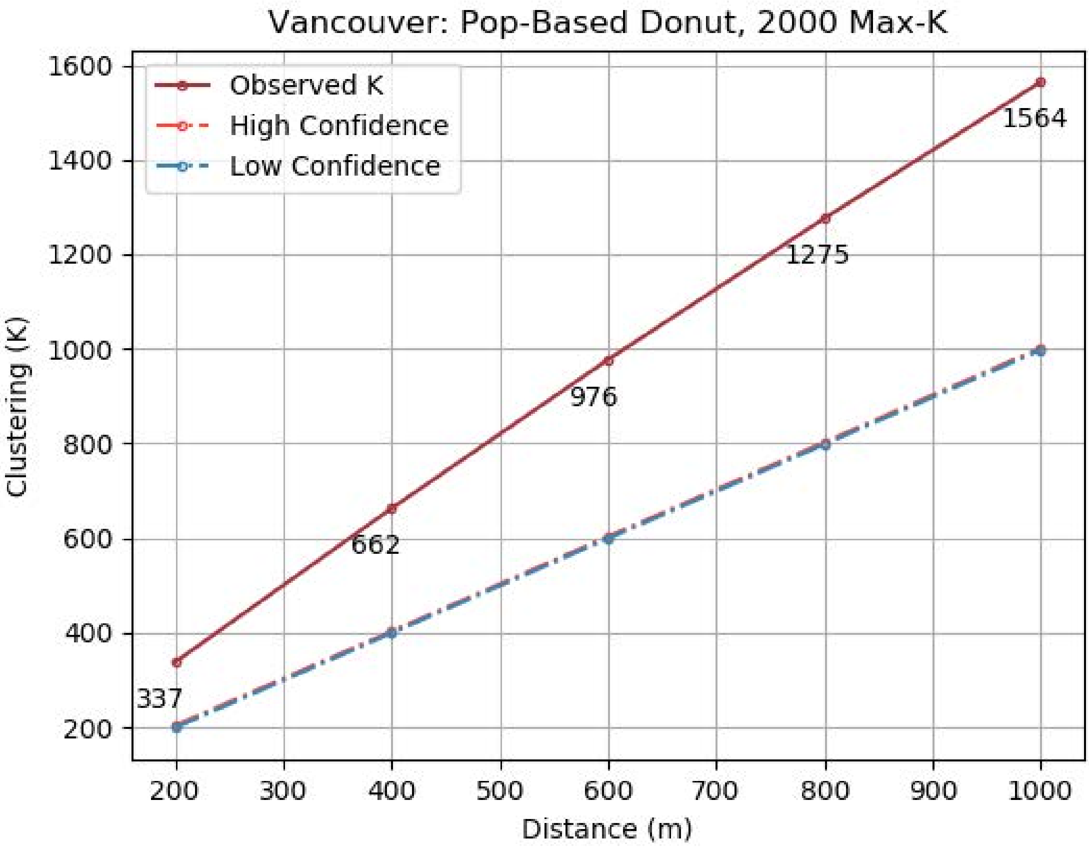

Vancouver: Pop-Based Donut, 2000 Max-K
Observed K
High Confidence
Low Confidence
Clustering (K)
Distance (m)
1600
1400
1200
1000
800
600
400
200
200
300
400
500
600
700
800
900
1000
337
662
976
1275
1564

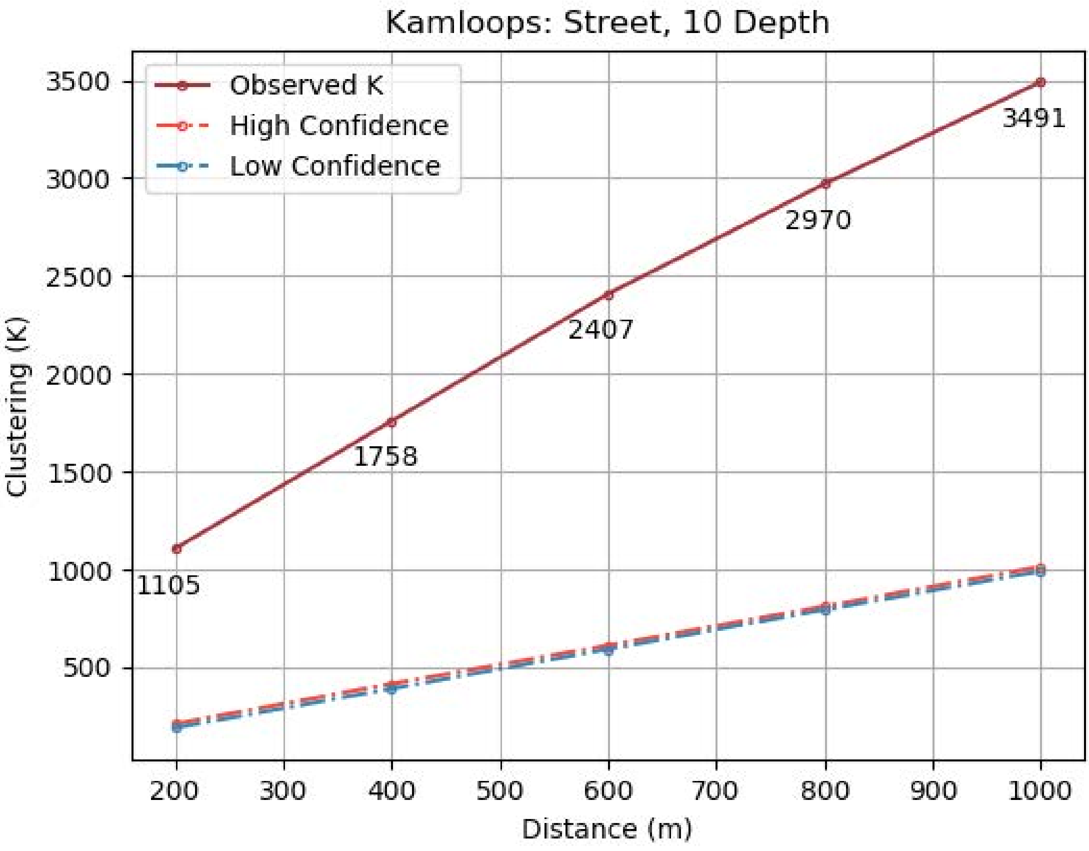
Kamloops: Street, 10 Depth
Observed K
High Confidence
Low Confidence
3500
3000
2500
2000
1500
1000
500
Clustering (K)
200
300
400
500
600
700
800
900
1000
Distance (m)
1105
1758
2407
2970
3491

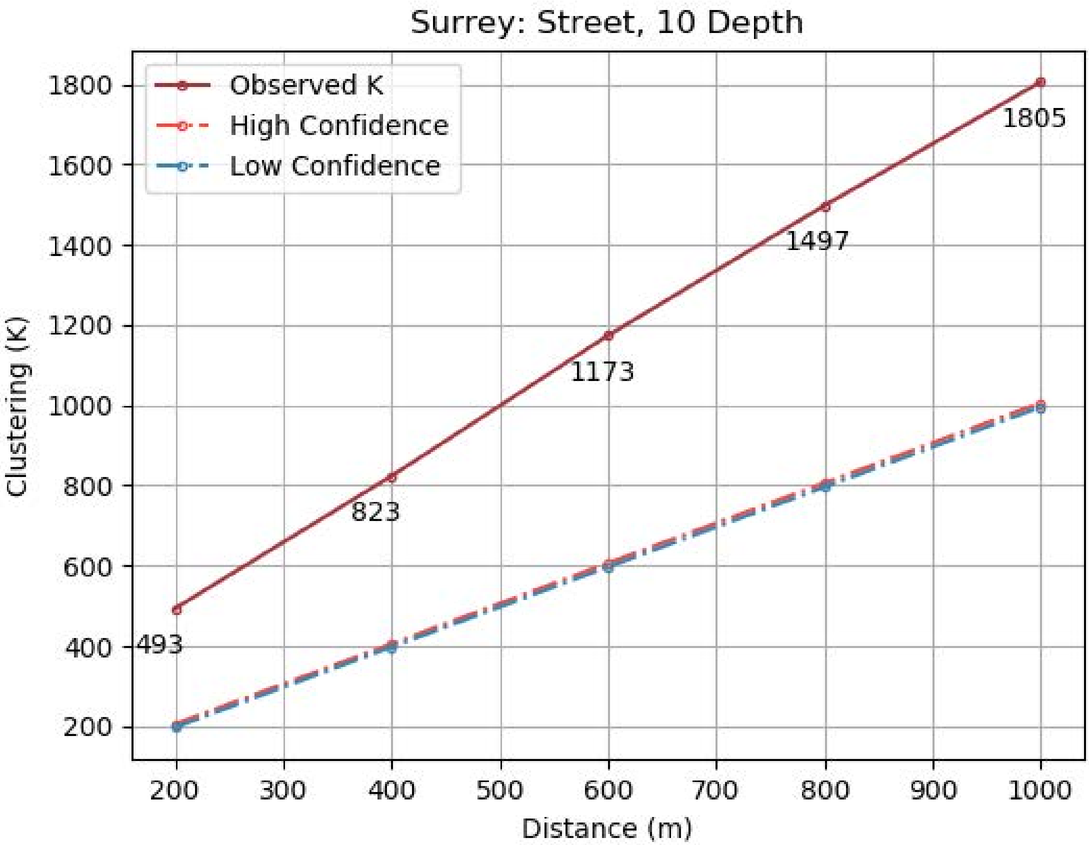

Surrey: Street, 10 Depth
Observed K
High Confidence
Low Confidence
Clustering (K)
Distance (m)
1805
1497
1173
823
493
200
400
600
800
1000
1200
1400
1600
1800
200
300
400
500
600
700
800
900
1000

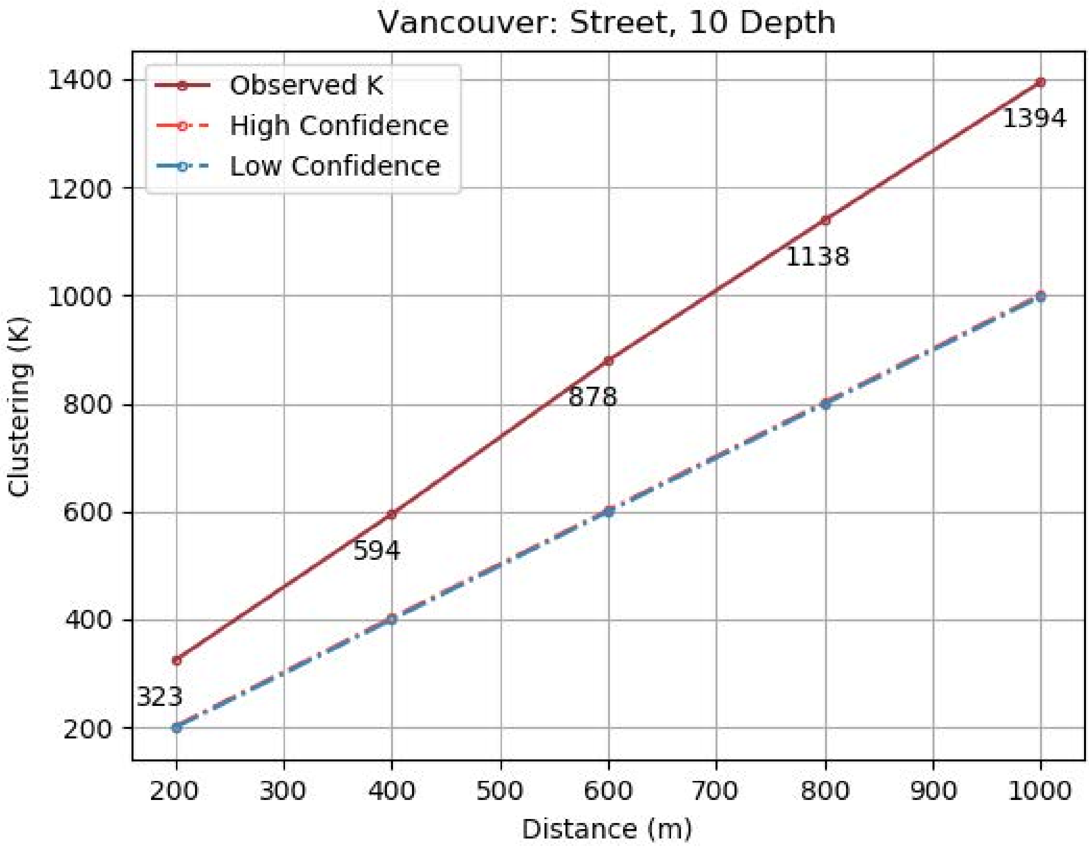

Vancouver: Street, 10 Depth
Observed K
High Confidence
Low Confidence
1394
1138
878
594
323
Clustering (K)
Distance (m)
1400
1200
1000
800
600
400
200
200
300
400
500
600
700
800
900
1000

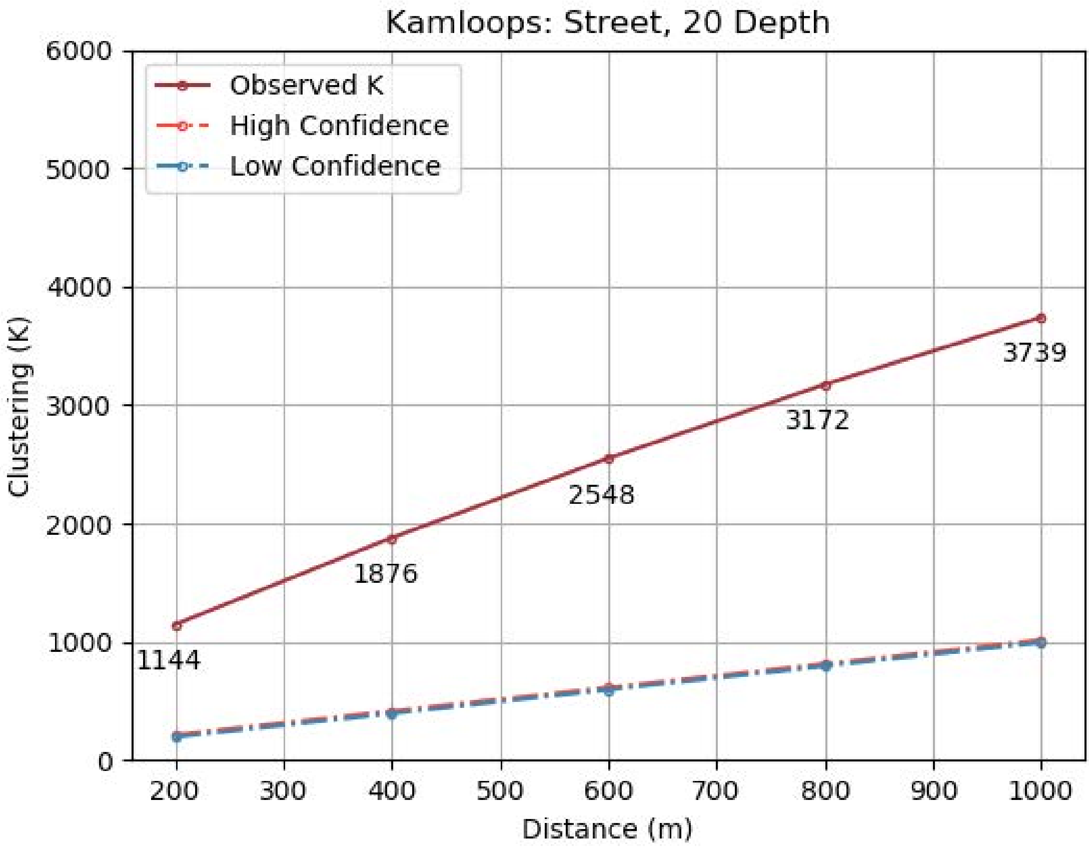
Kamloops: Street, 20 Depth
Observed K
High Confidence
Low Confidence
Clustering (K)
Distance (m)
0
1000
2000
3000
4000
5000
6000
200
300
400
500
600
700
800
900
1000
1144
1876
2548
3172
3739

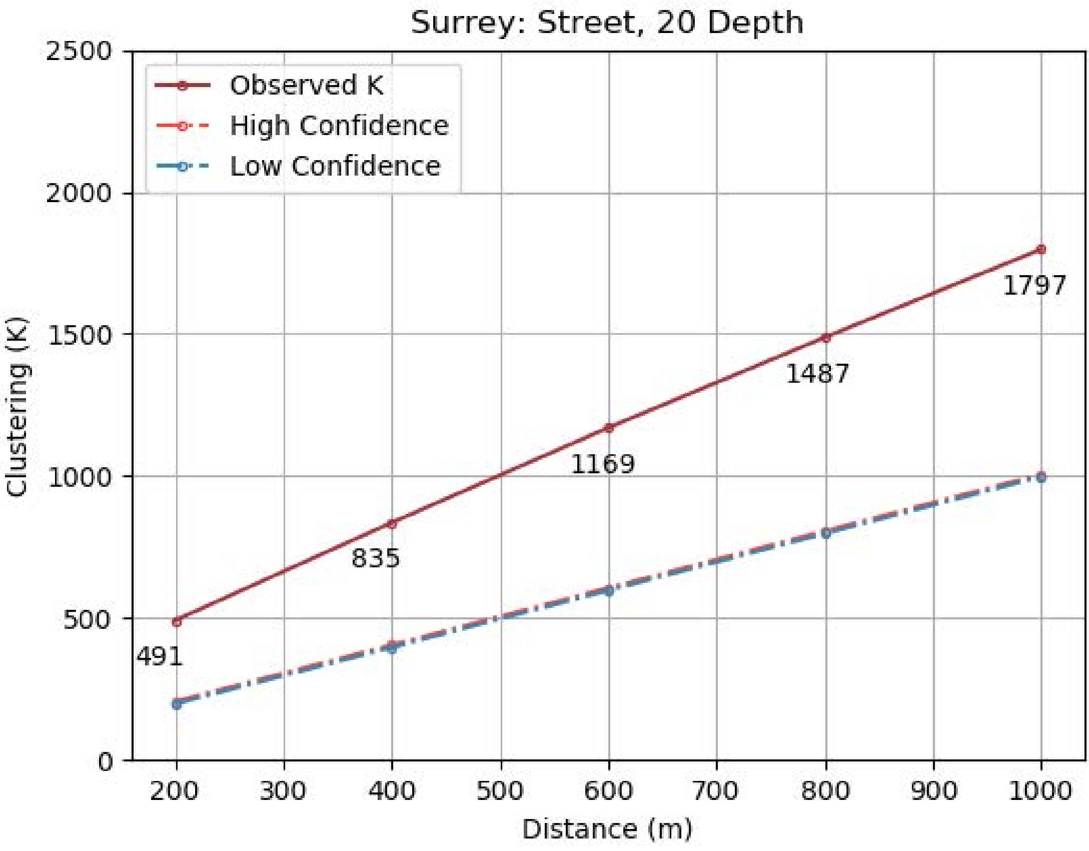

Surrey: Street, 20 Depth
Observed K
High Confidence
Low Confidence
Clustering (K)
Distance (m)
0
500
1000
1500
2000
2500
200
300
400
500
600
700
800
900
1000
491
835
1169
1487
1797

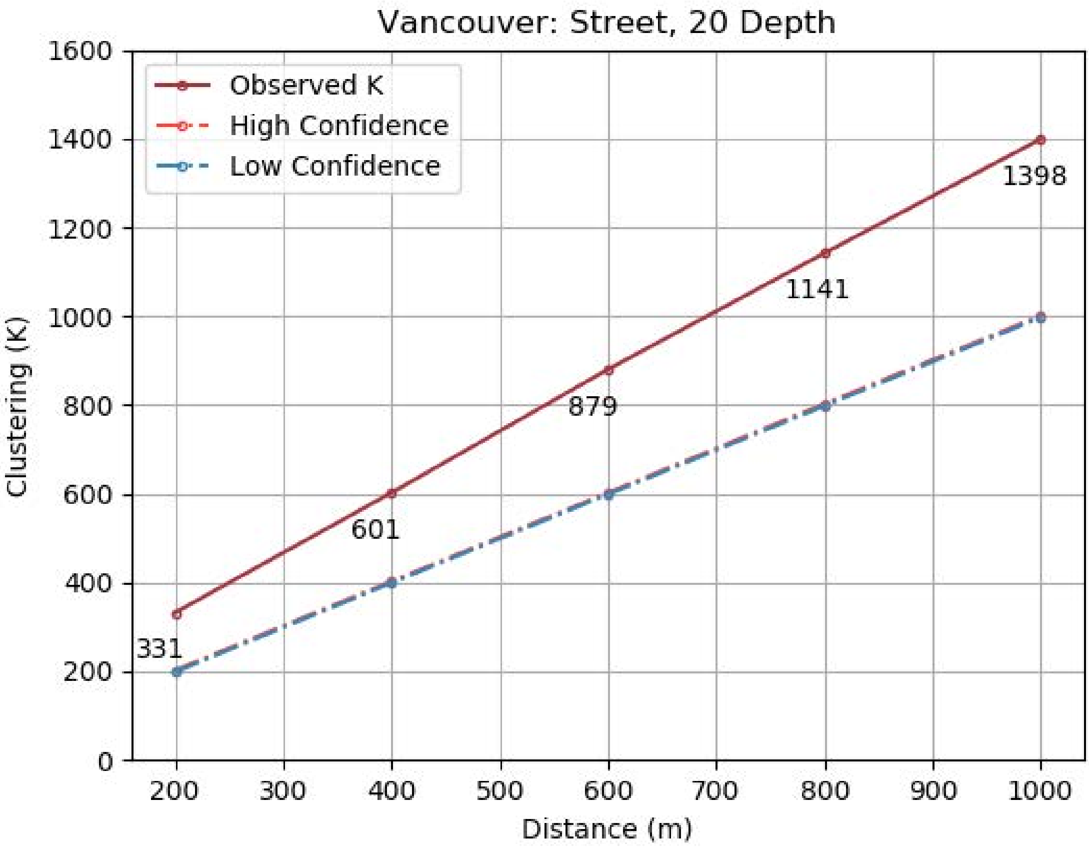
Vancouver: Street, 20 Depth
Observed K
High Confidence
Low Confidence
Clustering (K)
Distance (m)
0
200
400
600
800
1000
1200
1400
1600
200
300
400
500
600
700
800
900
1000
331
601
879
1141
1398

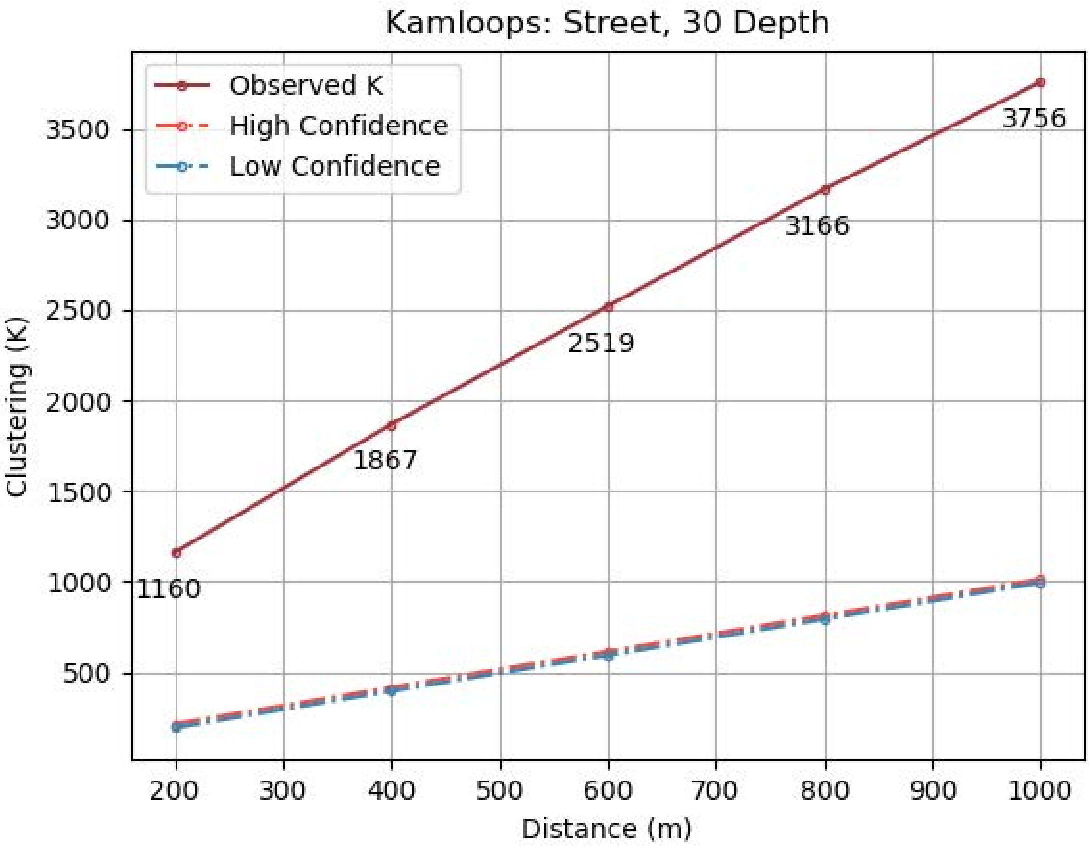
Kamloops: Street, 30 Depth
Observed K
High Confidence
Low Confidence
3756
3166
2519
1867
1160
Clustering (K)
3500
3000
2500
2000
1500
1000
500
200
300
400
500
600
700
800
900
1000
Distance (m)

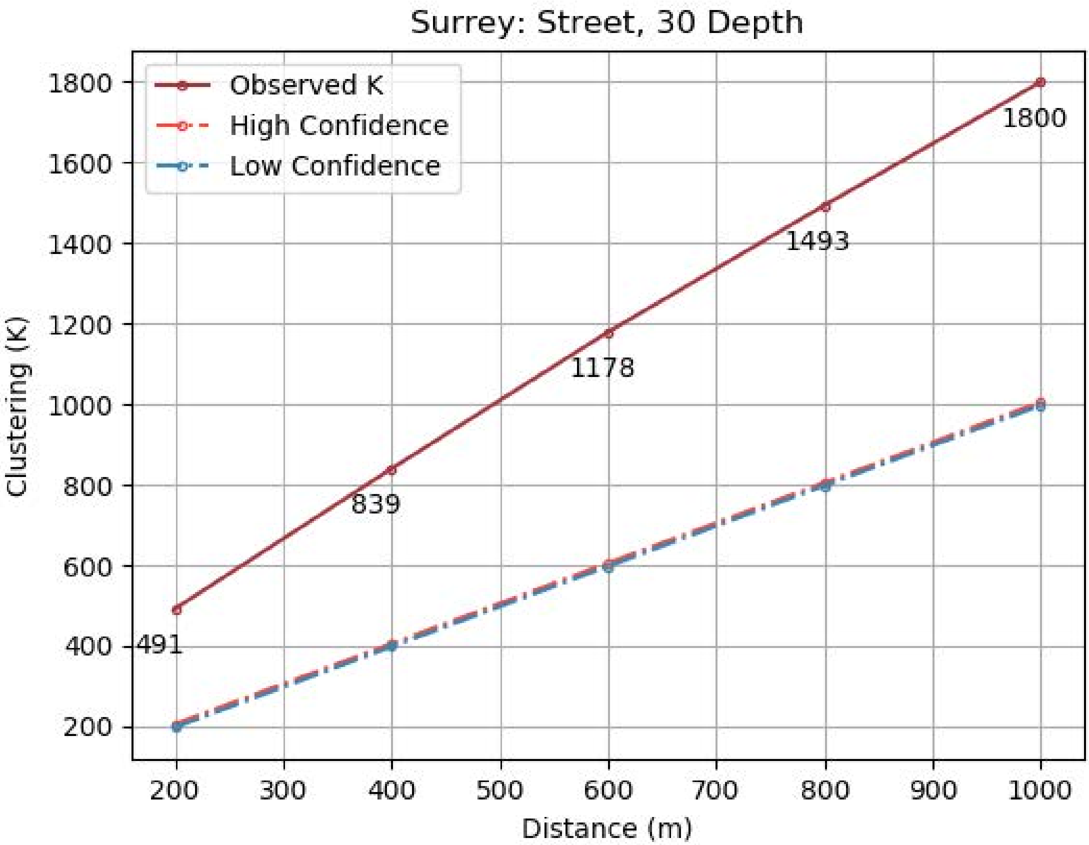

Surrey: Street, 30 Depth
Observed K
High Confidence
Low Confidence
1800
1493
1178
839
491
Clustering (K)
Distance (m)
200
300
400
500
600
700
800
900
1000
1600
1400
1200

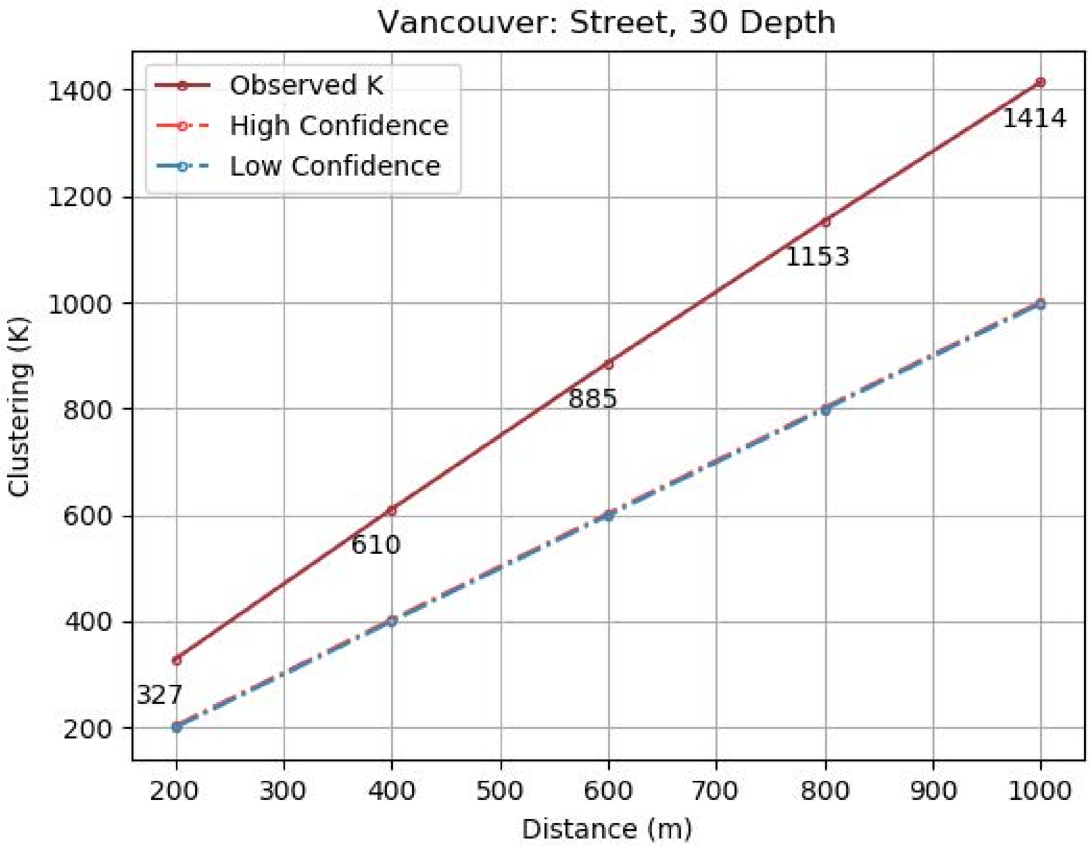

Vancouver: Street, 30 Depth
Observed K
High Confidence
Low Confidence
1414
1153
885
610
327
1400
1200
1000
800
600
400
200
Clustering (K)
200
300
400
500
600
700
800
900
1000
Distance (m)
